# Adaptive Bird-like Genome Miniaturization During the Evolution of Scallop Swimming Lifestyle

**DOI:** 10.1016/j.gpb.2022.07.001

**Published:** 2022-07-26

**Authors:** Yuli Li, Yaran Liu, Hongwei Yu, Fuyun Liu, Wentao Han, Qifan Zeng, Yuehuan Zhang, Lingling Zhang, Jingjie Hu, Zhenmin Bao, Shi Wang

**Affiliations:** 1 Sars-Fang Centre & MOE Key Laboratory of Marine Genetics and Breeding, College of Marine Life Sciences, Ocean University of China, Qingdao 266003, China; 2 Laboratory for Marine Biology and Biotechnology, Pilot Qingdao National Laboratory for Marine Science and Technology, Qingdao 266237, China; 3 Laboratory for Marine Fisheries Science and Food Production Processes, Pilot Qingdao National Laboratory for Marine Science and Technology, Qingdao 266237, China; 4 Key Laboratory of Tropical Aquatic Germplasm of Hainan Province, Sanya Oceanographic Institution, Ocean University of China, Sanya 572000, China; 5 CAS Key Laboratory of Tropical Marine Bio-resources and Ecology, South China Sea Institute of Oceanology, Chinese Academy of Sciences, Guangzhou 510301, China

**Keywords:** Genome size, Lifestyle evolution, Genome sequencing, Scallop, Bird

## Abstract

Genome miniaturization drives key evolutionary innovations of adaptive traits in vertebrates, such as the flight evolution of **birds**. However, whether similar evolutionary processes exist in invertebrates remains poorly understood. Derived from the second-largest animal phylum, **scallops** are a special group of bivalve molluscs and acquire the evolutionary novelty of the swimming lifestyle, providing excellent models for investigating the coordinated genome and **lifestyle evolution**. Here, we show for the first time that **genome sizes** of scallops exhibit a generally negative correlation with locomotion activity. To elucidate the co-evolution of genome size and swimming lifestyle, we focus on the Asian moon scallop (*Amusium pleuronectes*) that possesses the smallest known scallop genome while being among scallops with the highest swimming activity. Whole-**genome sequencing** of *A. pleuronectes* reveals highly conserved chromosomal macrosynteny and microsynteny, suggestive of a highly contracted but not degenerated genome. Genome reduction of *A. pleuronectes* is facilitated by significant inactivation of transposable elements, leading to reduced gene length, elevated expression of genes involved in energy-producing pathways, and decreased copy numbers and expression levels of biomineralization-related genes. Similar evolutionary changes of relevant pathways are also observed for bird genome reduction with flight evolution. The striking mimicry of genome miniaturization underlying the evolution of bird flight and scallop swimming unveils the potentially common, pivotal role of genome size fluctuation in the evolution of novel lifestyles in the animal kingdom.

## Introduction

Eukaryotic genomes vary greatly in size by more than five orders of magnitude [1], [2]. The variation of genome sizes caused by stochastic genetic processes has consequences for organismal fitness and, therefore, may act as the target of natural selection [2]. In vertebrates, genome size has been tentatively associated with life cycle complexity [3], metabolic rate [4], [5], body size [6], longevity [7], and developmental rate [8]. Particularly, flying creatures tend to have smaller genomes, which has been recognized as evidence of natural selection acting on genome size [5], [9]. The condition was construed as the pivotal adaptation of bird flight behavior by reducing the metabolic costs caused by large genome and cell size [9], [10]. In contrast to several well-known cases in vertebrates, the effect of genome size variation on invertebrate adaptive evolution remains largely unexplored [11], [12].

With more than 100,000 extant species, mollusca is the second-largest animal phylum, which globally distributes among diverse aquatic and terrestrial environments [13] and has succeeded in surviving through several mass extinction events [14]. The genome sizes of molluscs range from 293 Mb for *Neomenia permagna* in solenogastres to 7.68 Gb for *Diplommatina kiiensis* in gastropoda. Swimming is one of the special lifestyles among molluscs with calcified shells. Different from most bivalves with sessile and buried lifestyles, jet propulsion is produced by rapid and successive contraction of adductor muscle [15]. The swimming lifestyle of scallops is generally considered as an evolutionary novelty [16], representing excellent models for investigating the coordinated genome and lifestyle evolution. Distinct lifestyles from cementing, byssal attachment with sporadic swimming, recessing, and free-living to gliding (classified into ecomorphs A to E) have been well documented by measurement of muscle use in inducing escape responses [17], [18]. Scallops with gliding behavior are recognized as the most active swimmers. Shell morphology and metabolic capacity of adductor muscle are closely related to the swimming endurance of scallop species [17], [18]. However, genomic bases underlying the evolution of the scallop swimming lifestyle remain poorly understood. To date, the published scallop genomes are all derived from ecomorph B, C, or D [19], [20], [21], [22], [23], [24], and none of the genomes have been published for scallops with ecomorph E (gliding). The Asian moon scallop (*Amusium pleuronectes*; Linnaeus, 1758), a tropical Indo-West Pacific bivalve, is naturally distributed along the coast from Myanmar to Dampier Archipelago, north to southern Japan, and east to Papua New Guinea [15]. Thin and circular shells and gigantic adductor muscle make *Amusium* genus the most active scallops (belonging to the ecomorph E) [15], [25]. Decoding the genome from the scallop lineage with ecomorph E is crucial for accomplishing comparative genome analysis to unravel the genomic bases underlying the evolution of the swimming lifestyle in scallops.

Here, we show for the first time that the genome sizes of scallops exhibit a generally negative correlation with locomotion activity. Genome sequencing of *A. pleuronectes* and comparative genomic analyses reveal genome miniaturization and relevant gene pathways that contribute to the evolution of the swimming lifestyle, which strikingly mimics genomic changes underlying bird flight evolution and provides insights into the pivotal role of genome size fluctuation in the evolution of novel lifestyles in the animal kingdom.

## Results and discussion

### Negative correlation between genome size and swimming activity

**Figure 1:**
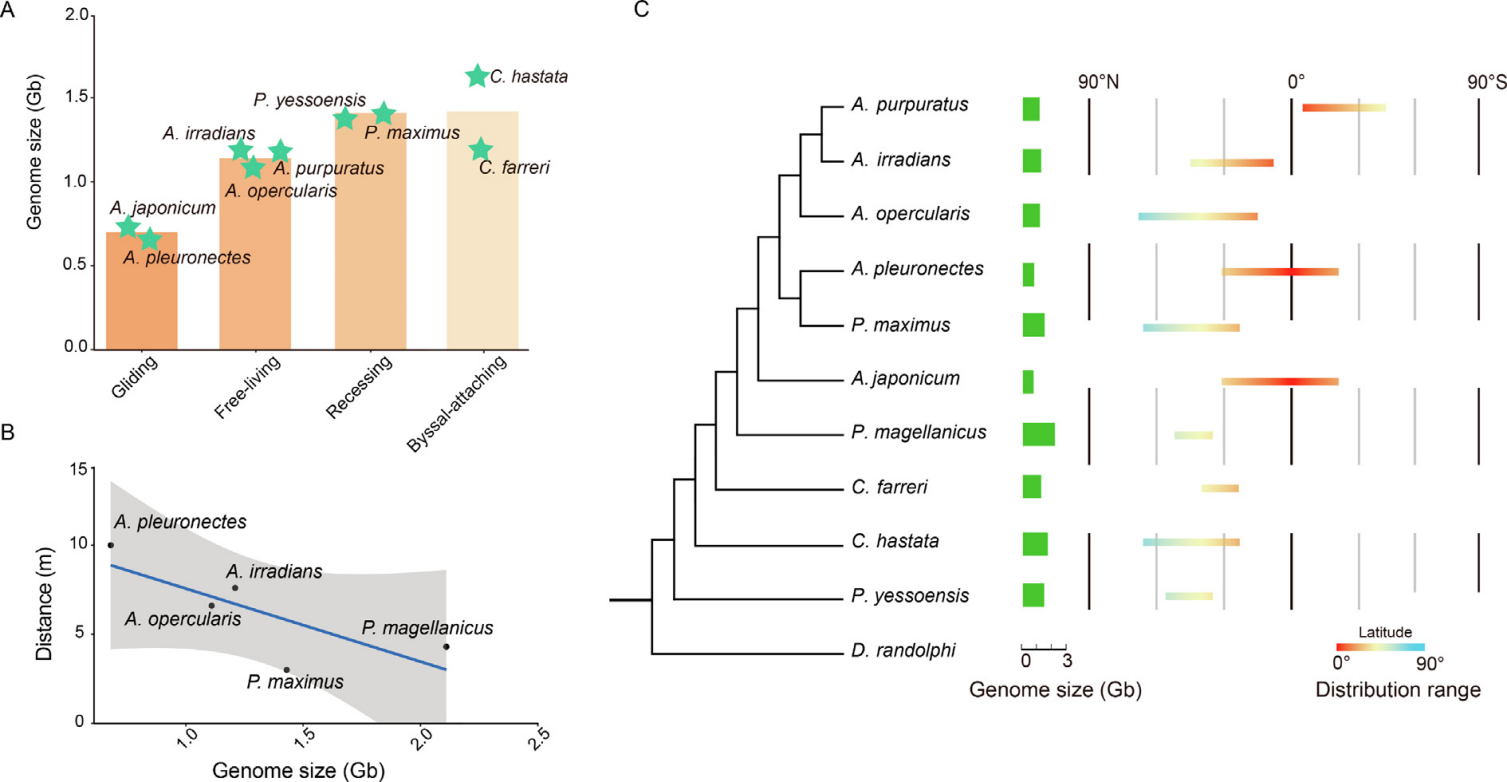
**Relationship of genome size, locomotion activity, and geographicaldistribution of scallops** **A.** Correlation of genome sizes of nine scallop species with different lifestyles from most inactive to most active (byssal-attaching, recessing, free-living, and gliding). **B.** Correlation of genome sizes with distances during one swimming burst for five scallop species. **C.** Correlation of genome sizes and geographical distributions for ten scallop species. *A. pleuronectes*, *Amusium pleuronectes*; *A. japonicum*, *Amusium japonicum*; *A. purpuratus*, *Argopecten purpuratus*; *A. irradians*, *Argopecten irradians*; *P. yessoensis*, *Patinopecten yessoensis*; *P. maximus*, *Pecten maximus*; *A. opercularis*, *Aequipecten opercularis*; *C. farreri*, *Chlamys farreri*; *C. hastata*, *Chlamys hastata*; *P. magellanicus*, *Placopecten magellanicus*; *D. randolphi*, *Delectopecten randolphi*.

Convergent and parallel evolution leads to various lifestyles in the family Pectinidae [16], [25]. The order of five lifestyles, ranging from cementing, byssal-attaching, recessing, and free-living to gliding, corresponds to the increase of scallop locomotion ability [16]. To explore the relationship of genome sizes with various lifestyles, we evaluated nine scallop species with genome size information from literature or animal genome size database (http://www.genomesize.com) and lifestyle classification (Table S1) according to the studies by Stanley and colleagues [26] and Serb and colleagues [25]. We found that scallop genome size substantially co-varied with lifestyle change (Figure 1A). Specifically, scallops with higher swimming activity possess smaller genome sizes, showing a negative correlation (*r* = −0.78) between genome size and swimming distance during one free swimming burst (Figure 1B; Table S2). For example, the gliding scallops of the *Amusium* genus, as the most active scallops [15], [17], possess the smallest genomes among the nine scallop species. Along with the decrease of motility from gliding to byssal-attaching, the genome size is gradually enlarged. Though our sampling number of scallop species is not very large but still comparable to those adopted in previous studies for investigating the relationship between genome size and biological traits [12], [27]. Such observation could be further strengthened by adopting more assayed scallop species in future studies.

The energy metabolism level and shell morphology are two important influencing factors on scallop locomotion activity [15], [17], [18]. The energy metabolism is closely related to the environmental temperature [28], [29]. We investigated the geographical distribution and environmental temperature of different scallops and found that scallops with smaller genomes reside at lower latitudes, such as the Asian moon scallops in the tropic ocean, while scallops with larger genomes, such as the Yesso scallop (*Patinopecten yessoensis*) and the Queen scallop (*Aequipecten opercularis*), reside at higher latitude oceans (Figure 1C; Tables S1 and S3). The shell morphology is predominantly associated with shell thickness and shell shape [15], [17]. In terms of shell thickness, the shells of Asian moon scallop are thinner and lighter [17], [25], and in terms of shell shape, the height/width ratio (∼ 1:1) of Asian moon scallop is more advantageous for swimming [17], [25]. To depict the genomic bases underlying the evolution of the scallop swimming lifestyle, we put a focus on the Asian moon scallop (*A. pleuronectes*) for genome sequencing and comparative analysis, due to its exhibition of the smallest genome with strikingly high swimming activity.

### The smallest but stable genome of ***A. pleuronectes***

**Figure 2:**
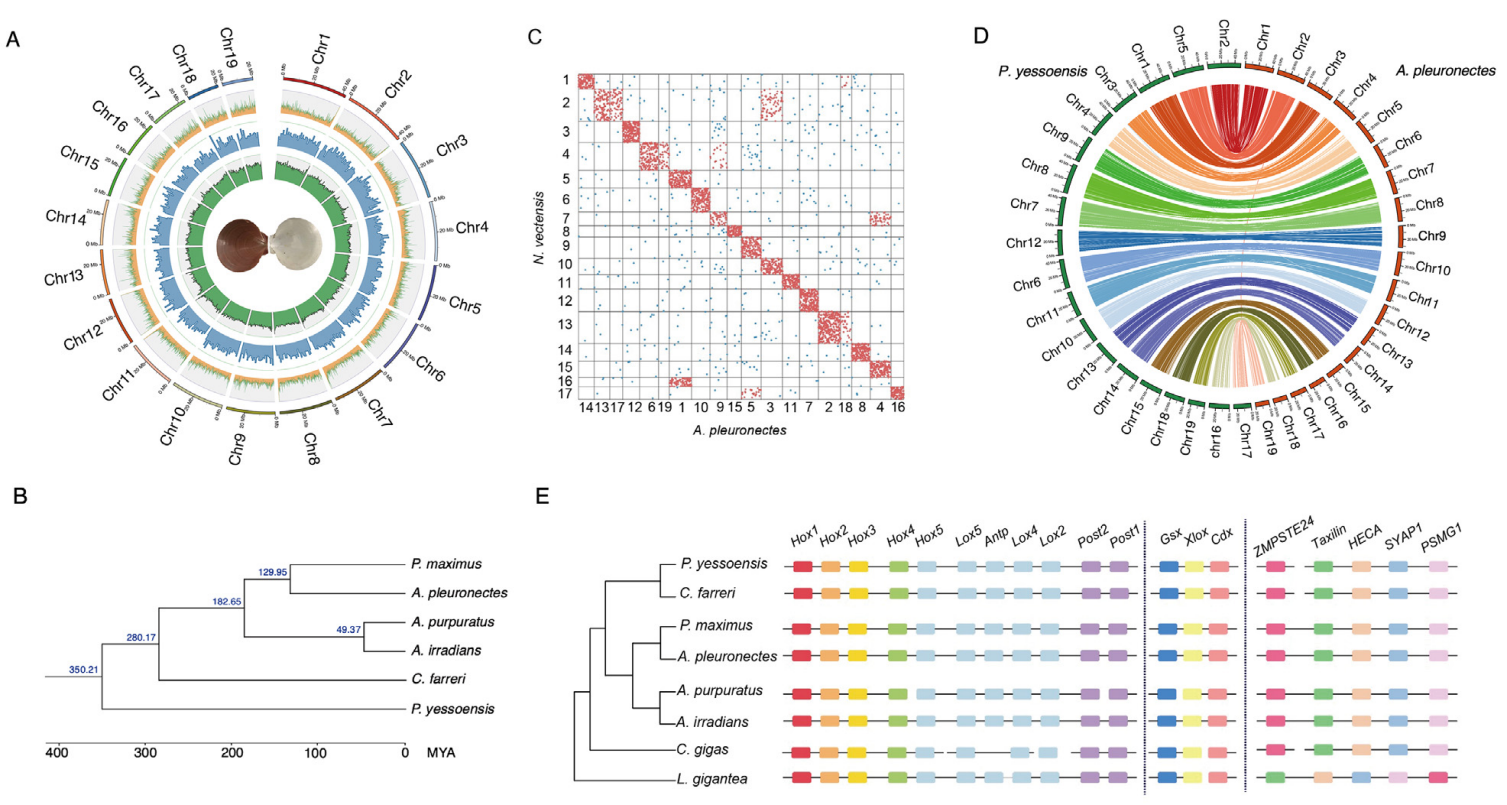
**Genome landscape, phylogenetic analysis, and gene synteny conservation of *A. pleuronectes*** **A.** Circos plot showing the genomic features of *A. pleuronectes*. The tracks from the outer to the inner indicate chromosome length, repeat sequence percentage in each 100 kb genomic interval, the density of gene distribution in each 1 Mb genomic interval, and GC content of 0.5 Mb genomic interval, respectively. **B.** Phylogenetic position of *A. pleuronectes* based on 4159 highly conserved, single-copy orthologous genes derived from six scallop species. The estimated divergence time is shown in blue on each branching point. **C.** Macrosynteny analysis of *A. pleuronectes* using *N*. *vectensis* as an ancestral linkage group reference. **D.** Macrosynteny between *A. pleuronectes* and *P. yessoensis.***E.** Microsynteny analysis of eight scallop species based on three conserved gene clusters. MYA, million years ago; *N. vectensis*, *Nematostella vectensis*; *C. gigas*, *Crassostrea gigas*; *L. gigantea*, *Lottia gigantea*.

**Supplementary Figure S1:**
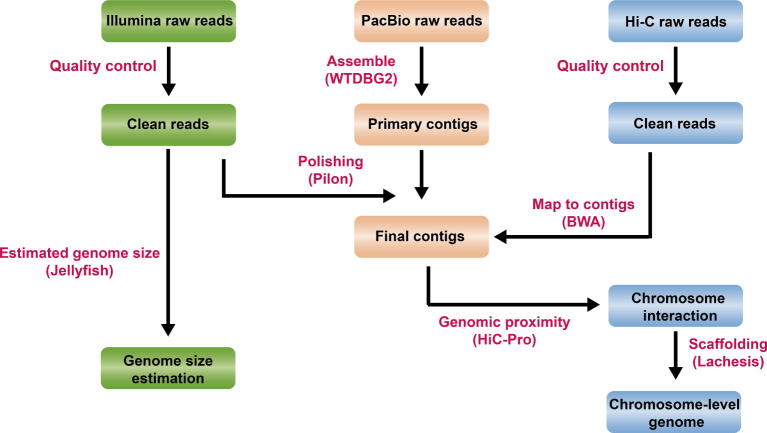
**The genome assembly pipeline**

**Supplementary Figure S2:**
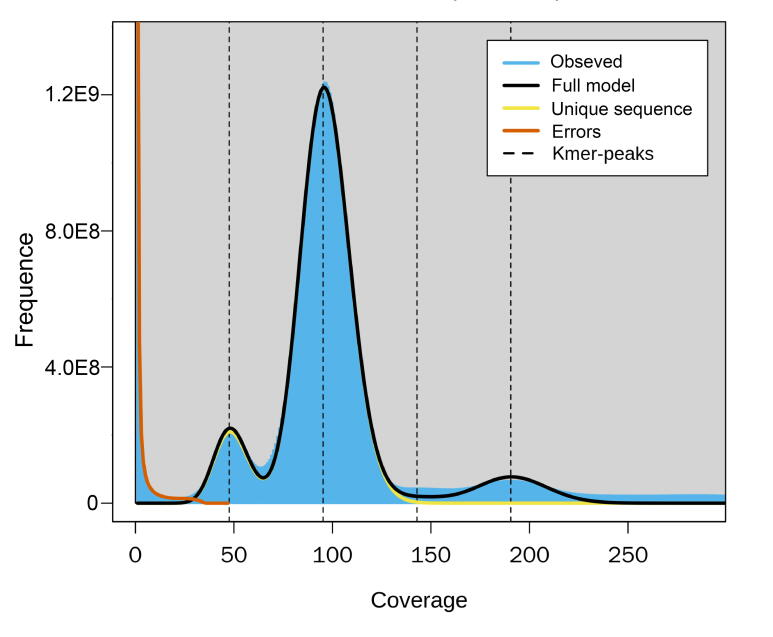
**Estimation of *A. pleuronectes* genome size based on genome survey data.** The three peaks are heterozygous, homozygous, and repeated 19-mers. The estimated heterozygosity of the genome is 0.60%, and the estimated genome size is 667.07 Mb.

**Supplementary Figure S3:**
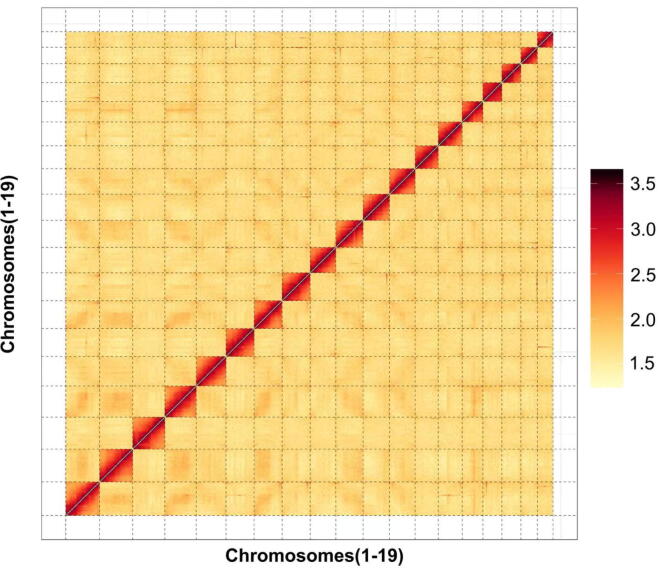
**Hi-C interaction heatmap of *A. pleuronectes* genome assembly showing contacts among 19 chromosomes**. The color reflects the intensity of each contact, and the darker the color, the stronger the interaction.

We conducted the sequencing and assembly of the smallest known scallop genome of *A. pleuronectes*, resulting in a total of 275.3 Gb of genomic data (Figure S1; Tables S4 and S5). The final genome assembly size is 626.63 Mb with contig N50 of 2.64 Mb and scaffold N50 of 34.61 Mb (Table S6), which is largely in accord with genome size estimated by *k*-mer analysis (667.07 Mb; Figure S2). A total of 609.55 Mb sequences (covering 97.27% of the total assembly) were anchored to 19 chromosomes (Figure 2A, Figure S3; Table S7), consistent with the dominant karyotypes of most scallops [19], [30]. We performed benchmarking universal single-copy orthologs (BUSCO) analysis for the quality assessment of genome assembly, obtaining 93.9% of complete and single-copy BUSCOs (Table S8).

Totally, 24,359 protein-coding genes were annotated, of which 95.34% (23,225 genes) were supported by at least one known protein sequence (Table S9). We also annotated 1872 non-coding RNAs (ncRNAs), containing 302 microRNAs (miRNAs), 908 transfer RNAs (tRNAs), 146 ribosomal RNAs (rRNAs), and 185 small nuclear RNAs (snRNAs) (Table S10). Furthermore, we identified 246.53-Mb repeat sequences (39.34% of the genome assembly), in which tandem repeats possess the highest proportion (21.7%), followed by DNA transposons (3.53%), long interspersed nuclear elements (LINEs, 2.91%), long terminal repeats (LTRs, 2.57%), and short interspersed nuclear elements (SINEs, 1.65%) (Table S11). Genome phylogeny analysis of six scallop species suggested that *A. pleuronectes* is most closely related to the king scallop *Pecten maximus* with a divergence time of about 130 million years ago (MYA) (Figure 2B).

**Supplementary Figure S4:**
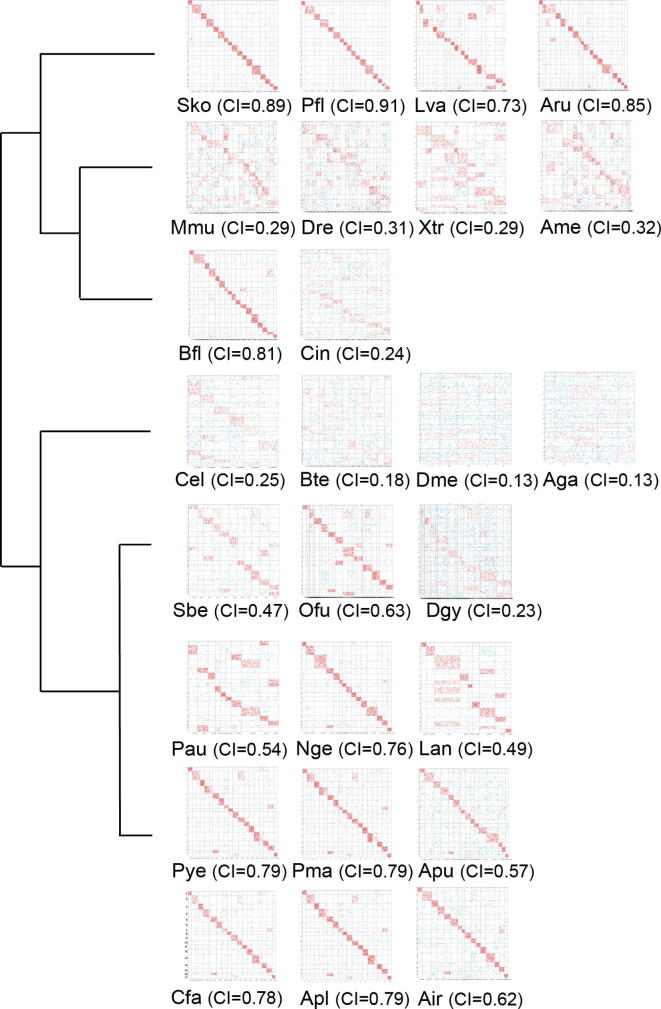
**Macrosynteny conservation of representative bilaterians genome** The dot plots show the conservation of gene macrosynteny between each species and the 17 bilaterian ALGs (*N. vectensis*), with red dots indicating significantly collinear chromosomes with *P*-value less than 0.05. Blue frames show the fusion of ALG 5 and ALG16. Ofu, *Owenia fusiformis*; Dgy, *Dimorphilus gyrociliatus*; Aga, *Anopheles gambiae*; Dme, *Drosophila melanogaster*; Pfl, *Ptychodera flava*; Xtr, *Xenopus tropicalis*; Ame, *Ailuropoda melanoleuca*; Bte, *Bombus terrestris*; Cel, *Caenorhabditis elegans*; Aru, *Asterias rubens*; Lva, *Lytechinus variegatus*; Bfl, *Branchiostoma floridae*; Sko, *Saccoglossus kowalevskii*; Mmu, *Mus musculus*; Dre, *Danio retio*; Cin, *Ciona intestinalis*; Lan, *Lingula anatina*; Nge, *Notospermus geniculatus*; Pau, *Phoronis australis*; Sbe, *Streblospio benedicti*.

The genome architecture of *A. pleuronectes* was further evaluated by gene synteny analysis. For macrosynteny analysis, we found that *A. pleuronectes* showed equivalent macrosynteny conservation levels with similar karyotypes and conservation index values in comparison with other scallops, including the *P*. *yessoensis* genome that was recognized with ancestral bilaterian karyotype [19] (Figure 2C and D, Figure S4). For microsynteny analysis, we investigated the conservation of the *Hox* and *ParaHox* gene clusters, which play crucial roles in the anterior-posterior body plan patterning during animal development [31]. The other gene cluster (*ZMPSTE24*-*Taxilin*-*HECA*-*SYAP1*-*PSMG1*) conserved in protostomes [32] was also checked (Table S12). We found that all three gene clusters were intact with conserved gene orders in the *A. pleuronectes* genome (Figure 2E). The high conservation of chromosomal macrosynteny and microsynteny implies the smallest *A. pleuronectes* genome was contracted but not degenerated during evolution.

### The smallest genome driven by transposon reduction

**Figure 3:**
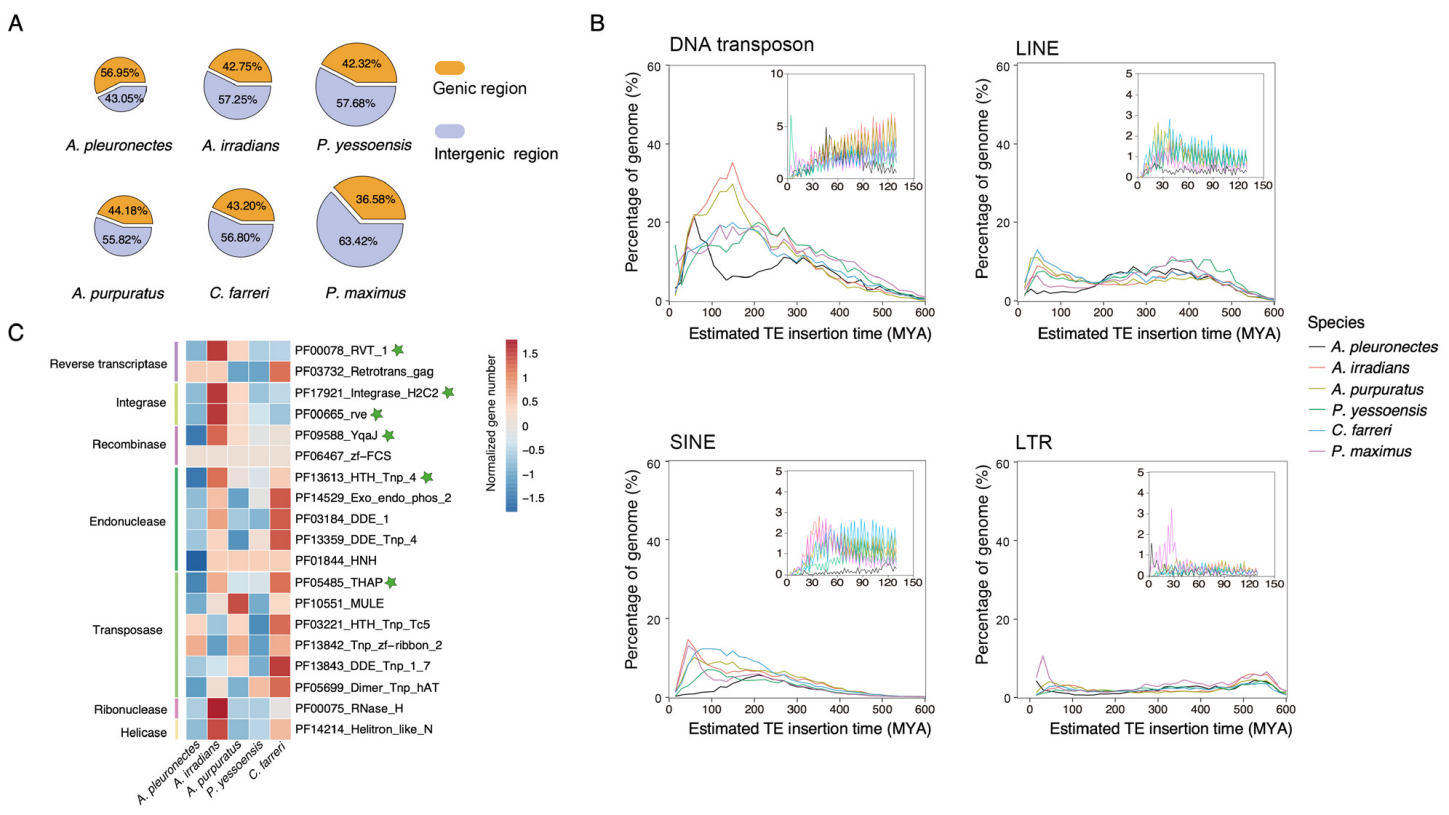
**Genome reduction of *A. pleuronectes* in comparison with other scallop species** **A.** Genome size comparison among six scallop species. **B.** Estimated insertion time of four TE types (DNA transposons, LINEs, LTRs, and SINEs) in the genomes of six scallop species. **C.** Comparison of the numbers of genes encoding transposition-related enzymes among different scallop species. The green star indicates the contracted gene families. TE, transposable element; LINE, long interspersed nuclear element; LTR, long terminal repeat; SINE, short interspersed nuclear element.

**Supplementary Figure S5:**
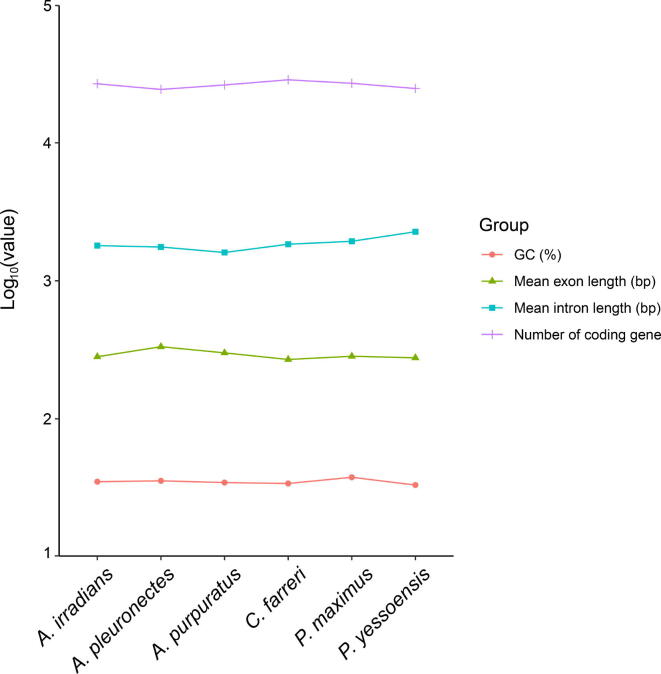
**Comparison of genomic architecture between six scallops (*A. irradians, A. purpuratus, A. pleuronectes, P. maximus, C. farreri*, and *P. yessoensis*)**

**Supplementary Figure S6:**
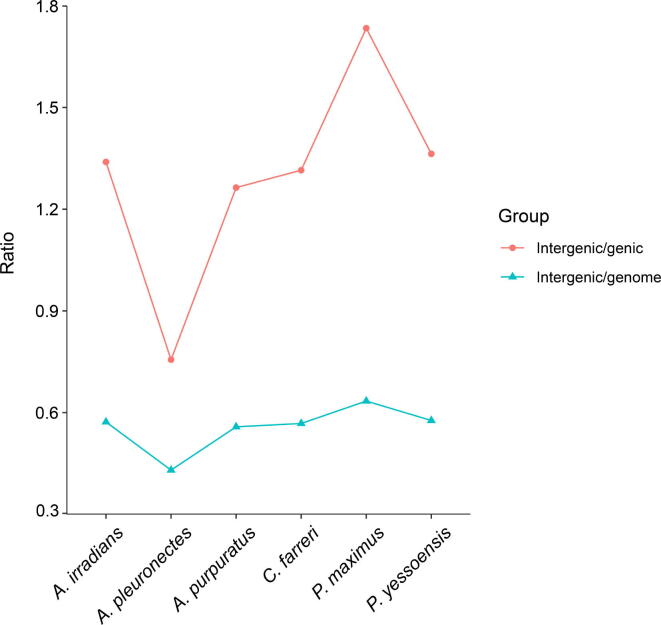
**Comparison of intergenic region/genome and intergenic region/genic region of six scallops (*A. irradians, A. purpuratus, A. pleuronectes, P. maximus, C. farreri,****and****P. yessoensis*)**

**Supplementary Figure S7:**
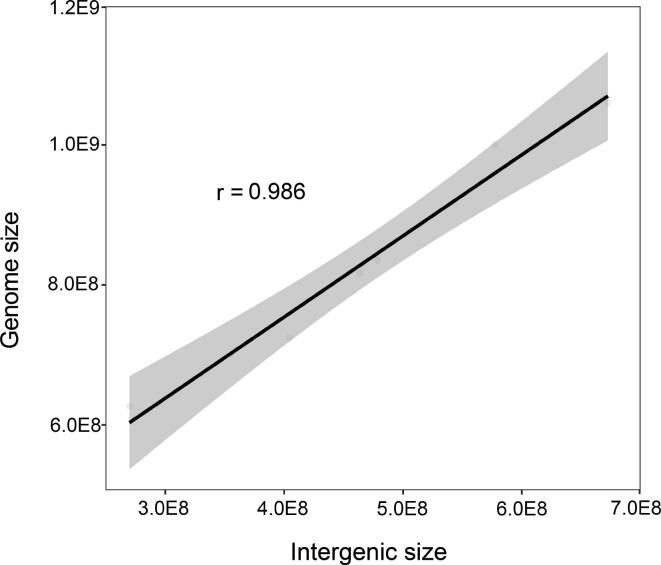
**Relationship between intergenic region size and genome size of six scallops (*A. irradians, A. purpuratus, A. pleuronectes, P. maximus, C. farreri,* and *P. yessoensis*)**

To depict the genome size evolution among scallop species, we conducted a comprehensive comparison of different genomic features for six scallop genomes that are currently available. Genomic architecture comparisons revealed that GC content, mean exon length, mean intron length, and coding gene number were similar among the six scallop species (Figure S5; Table S13). We found that the sizes of the genic regions among the six scallop species were all very close to the average size (average = 366 Mb, SD = 32 Mb), implying that the genic region kept stable during the diversification of scallop lineage. However, the sizes of the intergenic regions had a large variation among the six scallop species (SD= 126 Mb), and the *A. pleuronectes* genome possessed the smallest intergenic region and the smallest intergenic/genic ratio (Figure 3A, Figure S6). More importantly, the size of the intergenic region had a strong positive correlation with genome size (Figure S7), indicating that intergenic region size contributes greatly to the variation of scallop genome size.

**Supplementary Figure S8:**
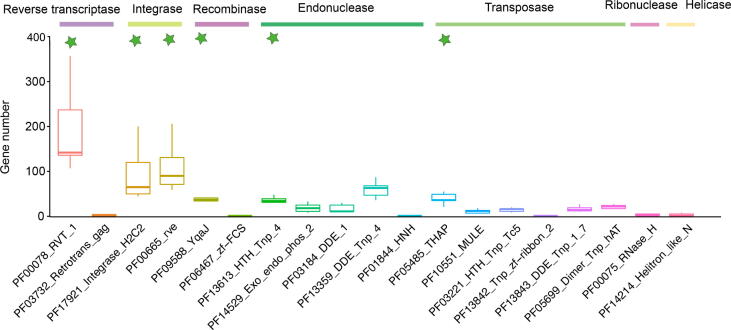
**Comparison of the gene numbers of different transposition-related enzyme families of scallops**

**Supplementary Figure S9:**
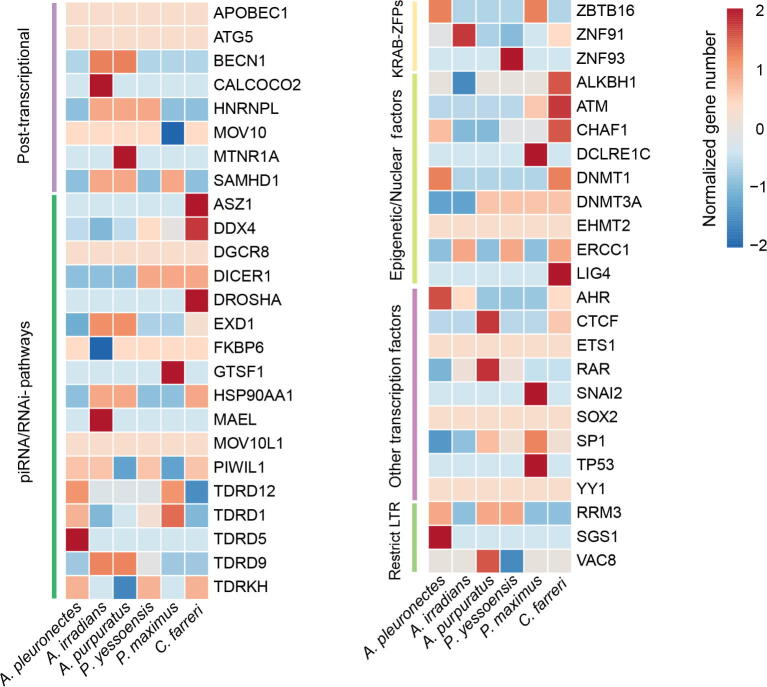
**Comparison of numbers of genes inhibiting transposon activity among scallops**

**Supplementary Figure S10:**
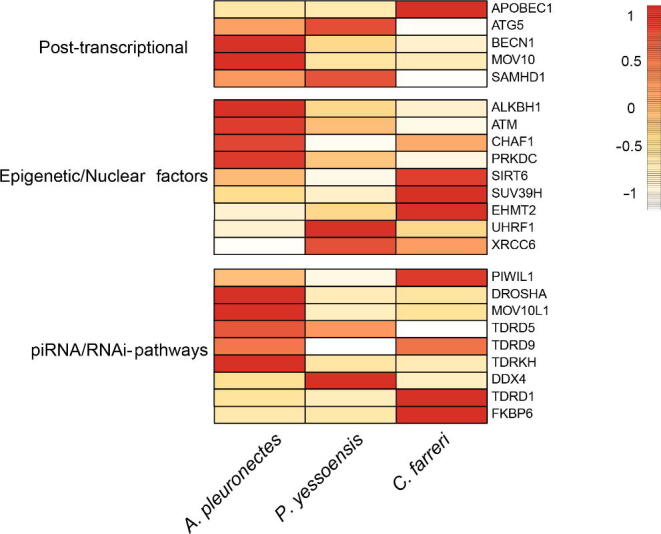
**Comparison for expression levels of genes inhibiting transposon activity among scallops**

We investigated the content variations of the two main repeat types [transposable elements (TEs) and tandem repeats] among scallop species, and found that TEs but not tandem repeats showed a significant reduction in *A. pleuronectes* genome (Table S13). Compared to *P. yessoensis* with the second-lowest TE contents, TEs in *A. pleuronectes* were reduced by 37.5% and 20% for the whole genome and intergenic regions, respectively. DNA transposons are the dominant TE type in scallop genomes, and we observed that *A. pleuronectes* genome lost most of the ancestral TEs that bursted about 150 MYA (Figure 3B). We found that TE expansion was remarkably inhibited in *A. pleuronectes* genome since barely any or little TE burst was observed after the divergence of *A. pleuronectes* with its closest relative *P. maximus* about 130 MYA. We further investigated the expansion/contraction of genes encoding transposition-related enzymes between *A. pleuronectes* genome and the other four scallop species by conducting a full search and comparison for genes encoding transposition-related enzymes. We found that for the several main classes (transposases, reverse transcriptases, integrases, recombinases, and endonucleases), the most dominant gene families with large gene numbers were contracted in *A. pleuronectes* genome (Figure 3C, Figure S8), including THAP domain-containing genes (PF05485) encoding proteins belonging to transposase, RVT_1 domain-containing genes (PF00078) encoding proteins belonging to reverse transcriptases, integrase_H2C2 (PF17921) and rve (PF00665) domain-containing genes encoding proteins belonging to integrases, YqaJ domain-containing genes (PF09588) encoding proteins belonging to recombinases, and HTH_Tnp_4 domain-containing genes encoding proteins belonging to endonucleases. We also investigated the expansion/contraction of genes that inhibit transposon activity among scallops [33], [34], but there were no significant trends for them (Figure S9). However, the activities of these genes represented by gene expression levels were elevated in *A. pleuronectes* (Figure S10). The TE reduction in non-coding regions of *A. pleuronectes* genome, caused by the contraction of genes encoding transposition-related enzymes and the elevated expression of TE inhibiting genes, leads to producing a “lighter” scallop genome that mimics flying birds [5], [9] and therefore paves the way for the evolution of high locomotion activity.

### Gene size reduction for high metabolic capacity in scallop and bird

**Figure 4:**
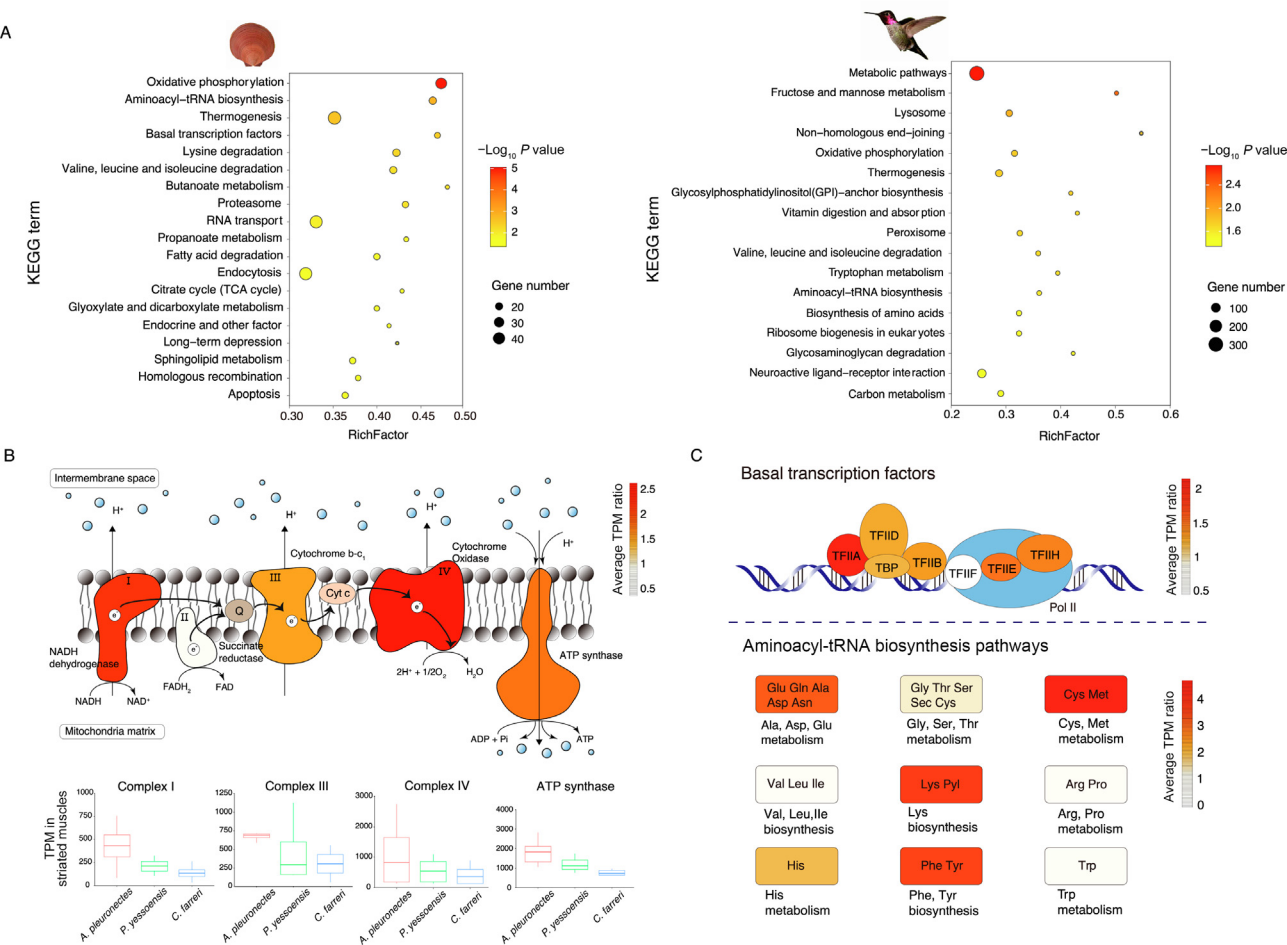
**Functional analysis and expressional comparison of size-reducing genes in scallop (*A. pleuronectes*) and bird (*C. anna*)** **A.** The KEGG enrichment analysis of size-reducing genes in the *A. pleuronectes* genome compared with five less-active scallop species (*P. yessoensis*, *A. purpuratus*, *A. irradians*, *P. maximus*, and *C. farreri*) and in the *C. anna* genome compared with three non-flying tetrapods (*C. serpentina*, *X. laevis*, and *M. musculus*). **B.** Comparison of expression levels (represented by TPM values) of size-reducing genes enriched in the oxidative phosphorylation pathway in the striated muscles of three scallop species (*A. pleuronectes*, *P. yessoensis*, and *C. farreri*). Colors of complexes I-IV and ATP synthase indicate the average TPM ratios in the striated muscle of *A. pleuronectes* to the other two scallop species. **C.** Comparison of expression levels of size-reducing genes enriched in the basal transcription factors and aminoacyl-tRNA biosynthesis pathways among four scallop species (*A. pleuronectes*, *P. yessoensis*, *C. farreri*, and *A. purpuratus*). Different colors indicate the average TPM ratios in *A. pleuronectes* to the other three scallop species. TPM, transcripts per million; *C. anna*, *Calypte anna*; *C. serpentina*, *Chelydra serpentina*; *X. laevis*, *Xenopus laevis*; *M. musculus*, *Mus musculus*.

**Supplementary Figure S11:**
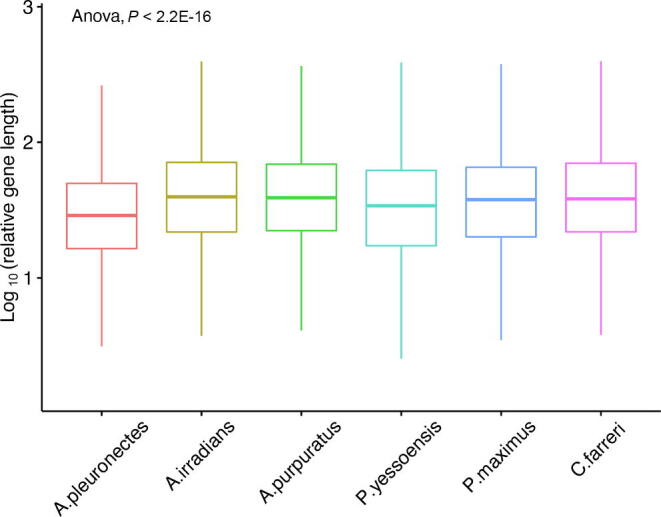
**Size-reducing genes in *A. pleuronectes* in comparison with the other five scallop genomes**

**Supplementary Figure S12:**
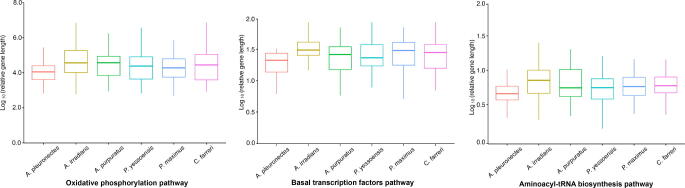
**Three significantly enriched pathways of size-reducing genes in the *A. pleuronectes* genome**

**Supplementary Figure S13:**
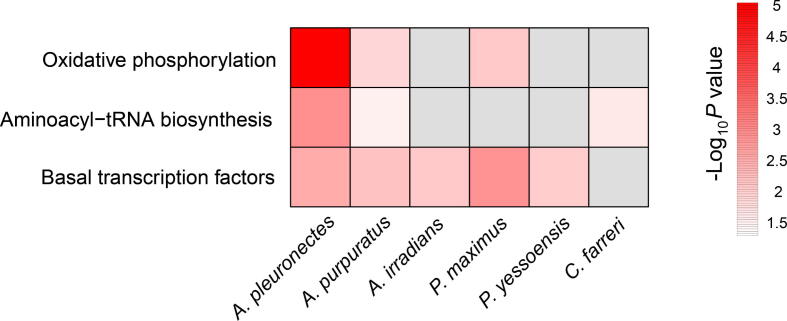
**Significance comparison of KEGG enrichment analyses of size-reducing genes for the three pathways among different scallop species**

**Supplementary Figure S14:**
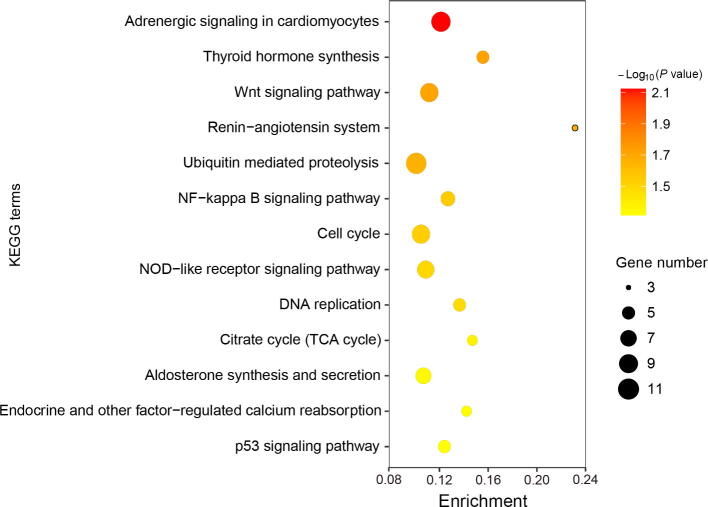
**KEGG enrichment analysis for positively selected genes in *A. pleuronectes***

The swimming behavior of scallops, which results from a rapid succession of adductor muscle contraction, is much related to the energy metabolism of the large striated muscle [17], [18]. Our group previously revealed that the large striated muscle of the scallop was energy-dynamic and showed higher energy demand in the swimming scallop than in the cementing oyster [20]. However, it remains unclear about the molecular driving forces that determine differential locomotion activities among different scallops. We conducted comprehensive genomic and transcriptomic analyses to depict the locomotion-related molecular changes underlying genome size reduction. From a genomic perspective, we identified 3794 size-reducing genes in *A. pleuronectes* when compared with the other five scallop species (Figure S11). Functional enrichment analysis of the size-reducing genes of *A. pleuronectes* revealed that these genes were most significantly enriched in the oxidative phosphorylation pathway (Figure 4A, Figures S12 and S13), which is the crucial energy-producing pathway responsible for the production of 95% ATP [35]. Particularly, we found that the TCA cycle, the other important part of aerobic respiration, was enriched in both size-reducing genes and positively selected genes (Figure S14), indicating the strong selective pressure on the energy metabolism process in *A. pleuronectes.* Moreover, these size-reducing genes were also found to be significantly enriched in basal transcription and translation processes (general transcription factors and aminoacyl-tRNA biosynthesis pathways) and basal metabolic pathways (Figure 4A, Figure S12). For the analysis of size-reducing genes in birds, we selected the hummingbird (*Calypte anna*), which is one of the fastest flying birds and possesses the highest metabolic rates normalized by body mass among vertebrates [36], [37]. We identified size-reducing genes in *C. anna* genome [38] (by comparing with other three selected tetrapods, including *Chelydra serpentina*, *Xenopus laevis*, and *Mus musculus*) and found that these genes were also significantly enriched in relevant pathways, including basal metabolic pathways and oxidative phosphorylation pathway (Figure 4A). These findings support the parallel evolution of gene size reduction for high metabolic capacity in both scallop swimming and bird flight.

**Supplementary Figure S15:**
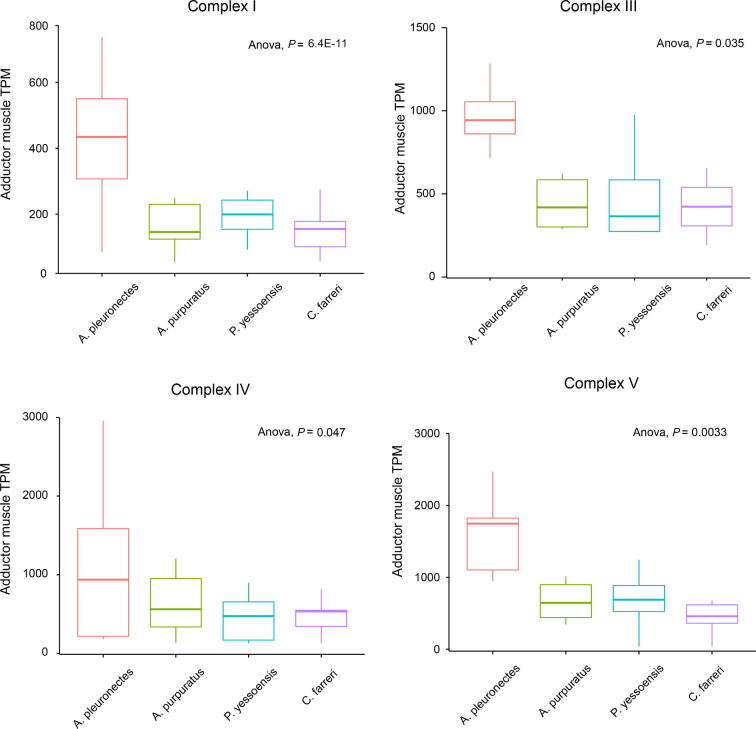
**Comparison for adductor muscle gene expression in oxidative phosphorylation pathway among the four scallop species**

Gene length is one of the well-known determining factors for transcriptional regulation, generally with faster and accumulated higher expression of shorter genes than longer genes [39]. To evaluate the transcriptional effect of size-reducing genes, we obtained the expression levels of these genes in three scallop species with different motility classifications (from least active ecomorph A to most active ecomorph E) and abundant adult tissue/organ transcriptomic data enabling for a comparative analysis. The selected scallop species were classified into three ecomorph types, including the most active scallop *A. pleuronectes* (ecomorph E), the recessing scallop *P. yessoensis* (ecomorph D), and the byssal-attaching scallop *Chlamys farreri* (ecomorph B). We compared the gene expression profiles in the striated muscles of three scallop species for the three gene pathways that were most enriched with size-reducing genes in *A. pleuronectes* (Figure 4B and C). For the energy-producing oxidative phosphorylation pathway, all genes showed higher expression in the striated muscle of *A. pleuronectes* than the other scallops (Figure 4B), particularly for complexes I, III, and IV, which contribute to the production of the proton gradient, and ATP synthase (complex V), which drives ADP phosphorylation to ATP (Figure 4B). We also mixed the transcriptome data of striated and smooth muscles of *A. pleuronectes*, *P. yessoensis*, and *C. farreri* to enable the comparison with the adductor muscle transcriptome data of *Argopecten purpuratus*, which still confirmed our previous finding that *A. pleuronectes* has higher expression levels in the oxidative phosphorylation pathway than the other three species (Figure S15). For the two basal cellular processes, we also found a significant increase of gene expression in *A. pleuronectes* than in the other three scallops (Figure 4C). These transcriptional changes manifest the functional role of gene size reduction for the adaptive evolution of high metabolic capacity in *A. pleuronectes*.

### Parallel repression of biomineralization genes in scallop and bird

**Figure 5:**
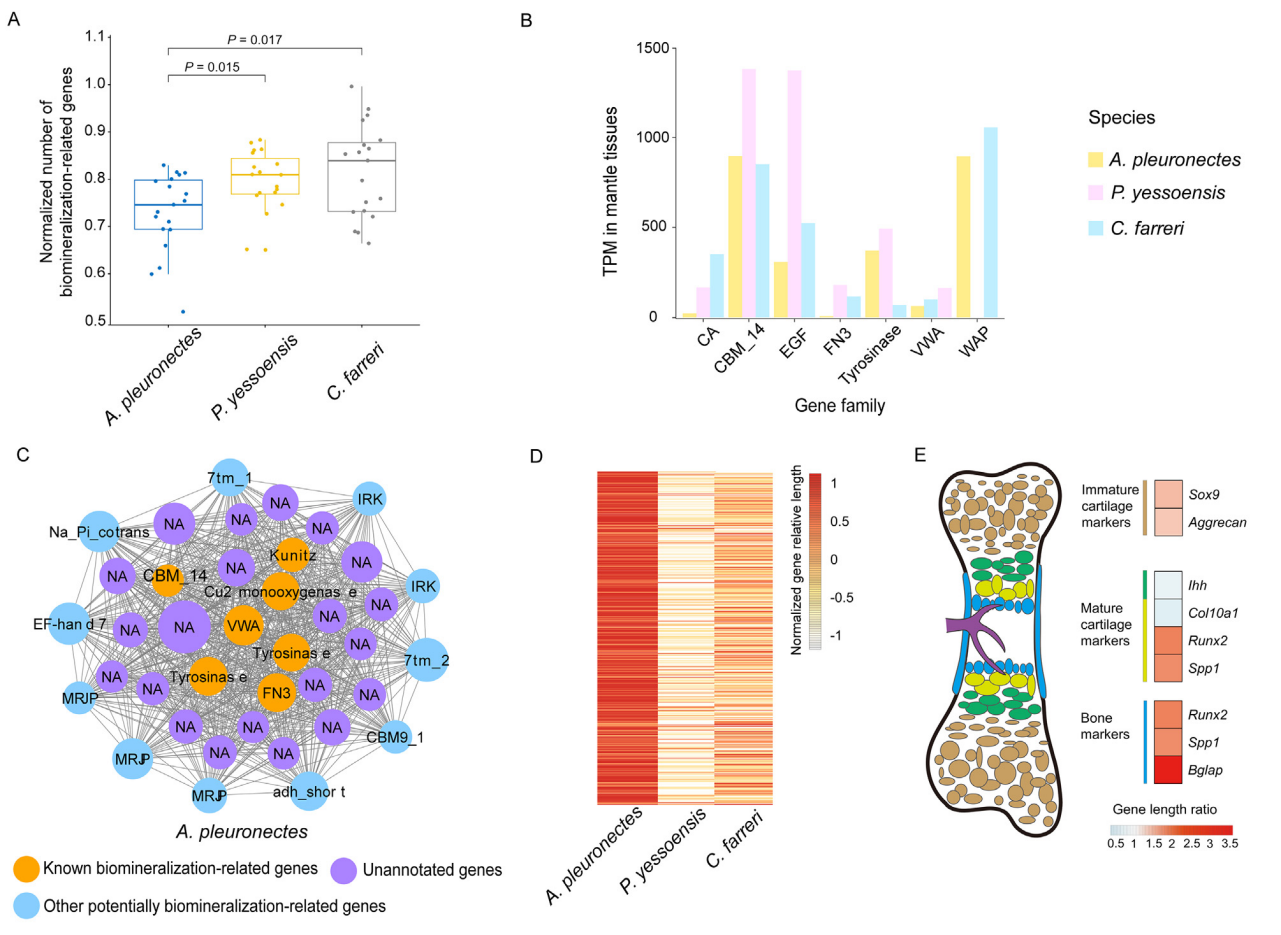
**Analysis of biomineralization-related genes in scallop (*A. pleuronectes*) and bird (*C. anna*)** **A.** Comparison of biomineralization-related genes between *A. pleuronectes* and the other two scallop species. The boxplot shows the copy number variations of known biomineralization-related genes in bivalves. **B.** The histogram shows the cumulative gene expression levels (represented by TPM values) of seven main biomineralization-related gene families in the mantle tissues of three scallop species. **C.** Biomineralization-related module of *A. pleuronectes*. **D.** Relative gene length comparison of 440 biomineralization-related genes by *de novo* search among three scallop species. **E.** Comparison of bone marker genes in flying birds and non-flying tetrapods. Different colors of genes show the ratio of gene length in the flying bird (*C. anna*) to the average gene length in the other three tetrapods (*C. serpentina*, *X. laevis*, and *M. musculus*). CA, carbonic anhydrase; CBM_14, chitin-binding peritrophin-A domain; EGF, epidermal growth factor; FN3, fibronectin-3 domain; VWA, von Willebrand factor type A; WAP, whey acidic protein.

**Supplementary Figure S16:**
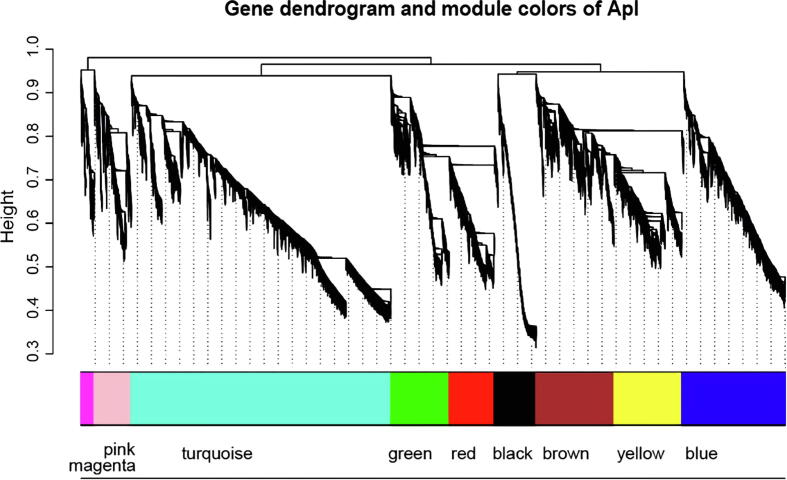
**Construction of gene coexpression network of *A. pleuronectes* based on 20 adult organ/tissue transcriptomes**

**Supplementary Figure S17:**
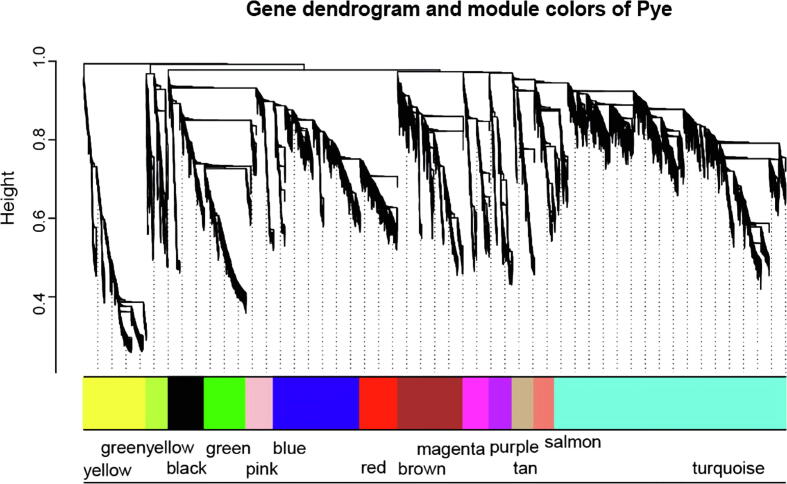
**Construction of gene coexpression network of *P. yessoensis* based on 28 adult organ/tissue transcriptomes**

**Supplementary Figure S18:**
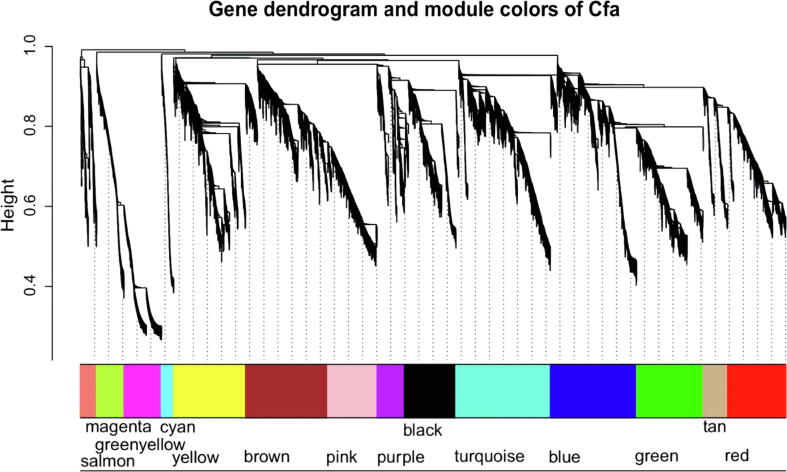
**Construction of gene coexpression network of *C. farreri* based on 32 adult organ/tissue transcriptomes**

**Supplementary Figure S19:**
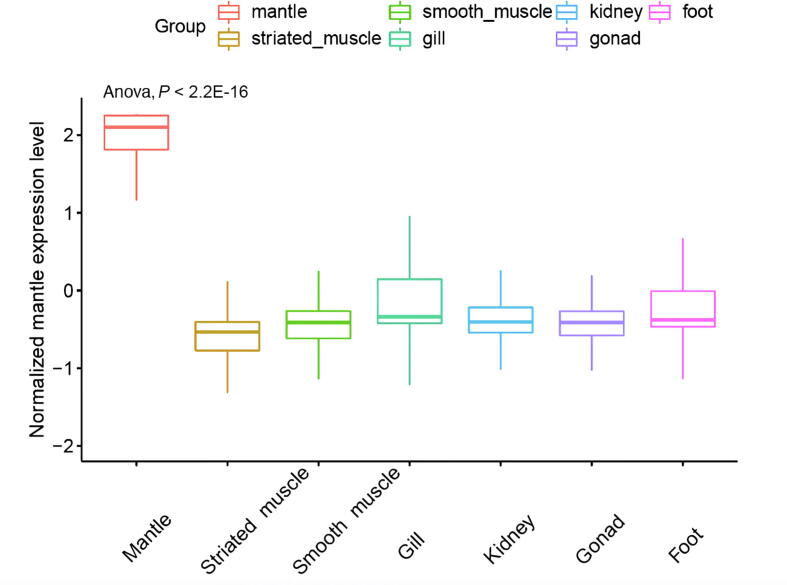
**Mantle expression level (normalized TPM) of biomineralization-related module in *A. pleuronectes***

**Supplementary Figure S20:**
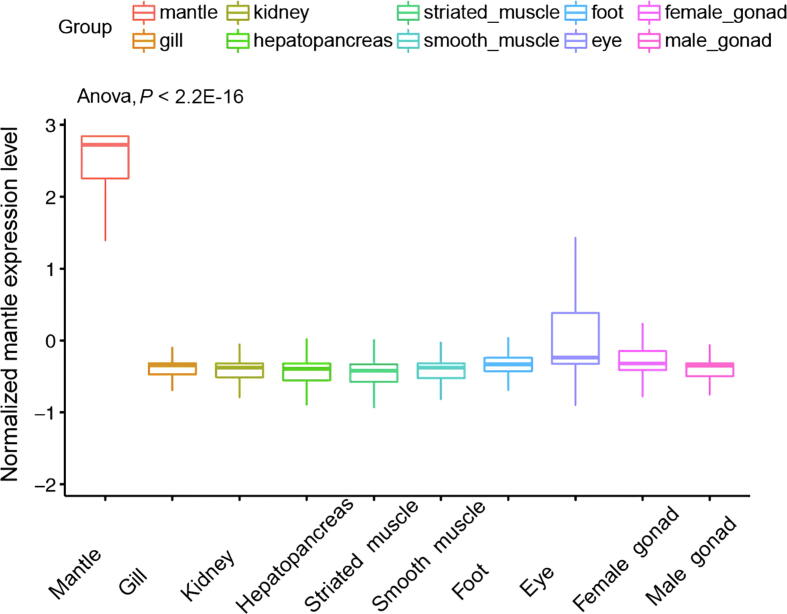
**Mantle expression level (normalized TPM) of biomineralization-module in *P. yessoensis***

**Supplementary Figure S21:**
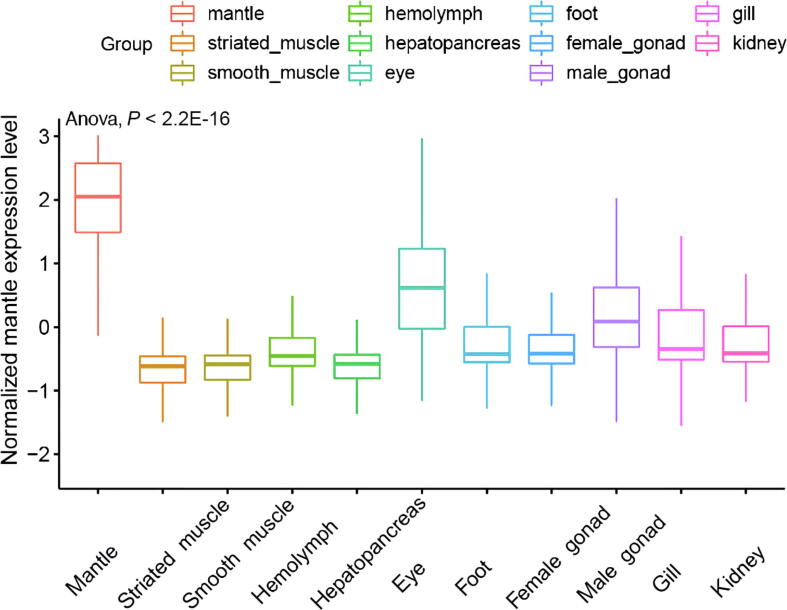
**Mantle expression level (normalized TPM) of biomineralization-module in *C. farreri***

**Supplementary Figure S22:**
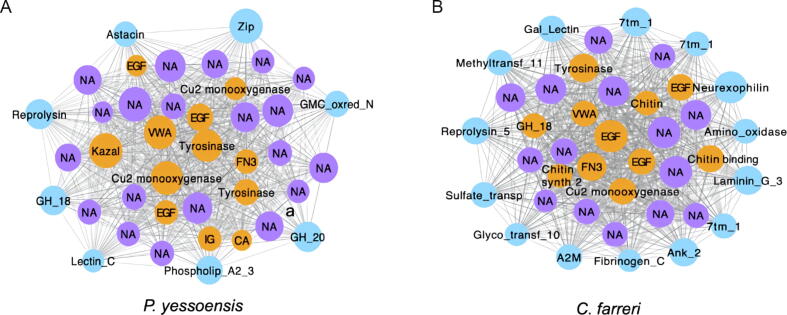
**Biomineralization-related modulesof *P. yessoensis* and *C. farreri***

**Supplementary Figure S23:**
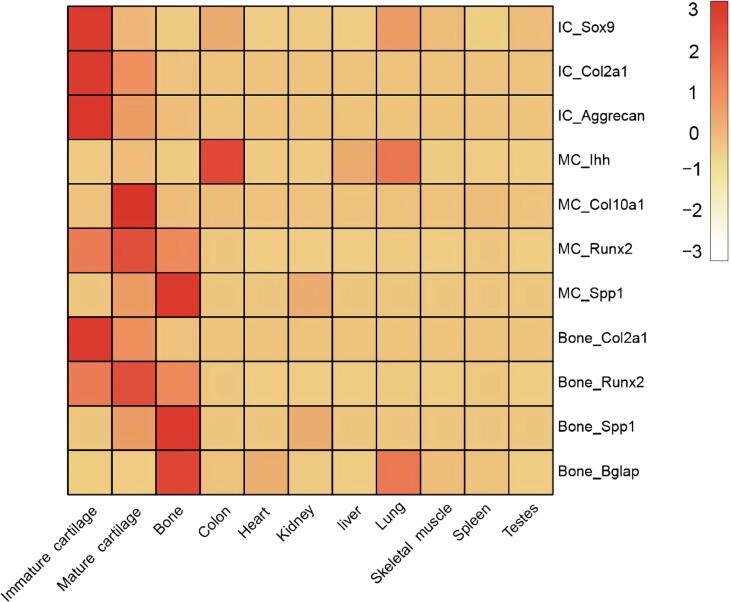
**Expression level (TPM) of bone marker genes in *Gallus gallus***

It has been reported that shell structure and weight are closely related to scallop locomotion activity [17]. There are many known biomineralization-related genes in bivalves, such as those encoding carbonic anhydrase (CA), tyrosinase, epidermal growth factor (EGF), von Willebrand factor type A (VWA), immunoglobin (IG), and fibronectin-3 domain (FN3) [40], [41]. We then investigated the copy numbers and expression profiles of these biomineralization-related genes (Table S14) in three scallop species, including *A. pleuronectes* (ecomorph E), *P. yessoensis* (ecomorph D), and *C. farreri* (ecomorph B). We found that most of the biomineralization-related genes have fewer copy numbers in *A. pleuronectes* than those in the other scallops (Figure 5A). The gene expression profiles of seven main gene families, including CA, chitin-binding peritrophin-A domain (CBM_14), EGF, FN3, tyrosinase, VWA, and whey acidic protein (WAP), showed lower expression levels in the mantle transcriptome of *A. pleuronectes* than in other scallop species (Figure 5B). To reveal the core biomineralization-related genes and their interaction relationships, we constructed the weighted correlation network analysis (WGCNA) gene co-expression networks (Figures S16–S18) and identified the biomineralization-related modules for *A. pleuronectes*, *P. yessoensis*, and *C. farreri*, in which the mantle-specific highly-expressed genes were significantly enriched (Figures S19–S21). As expected, several known biomineralization-related genes were found as hub genes (the top 40 genes ranked by intramodular connectivity) in the biomineralization-related modules of these three scallop species (Figure 5C, Figure S22). Notably, this biomineralization-related module was significantly enriched with the size-increasing genes identified in *A. pleuronectes* compared with the other two scallop species (*P* < 0.05, Figure 5D). The decrease in gene copy numbers or expression levels and increase in gene length of biomineralization-related genes in the Asian moon scallop may explain their thinner and lighter shells for more powerful motility than other scallop species. Interestingly, we also found a similar tendency in flying birds in comparison with other tetrapods, that is, the length of most bone marker genes [42] relatively increased in flying birds (Figure 5E, Figure S23), implying the convergent evolution of the depressed biomineralization process for acquisition of powerful motility in both vertebrates and invertebrates.

## Conclusion

We report for the first time that genome sizes of scallops show a generally negative correlation with locomotion activity. Sequencing of the smallest genome of Asian moon scallop *A. pleuronectes* enables the decoding of co-evolution of genome size and swimming lifestyle. Transposon reduction in the *A. pleuronectes* genome, resulting from significant contraction of several transposon-related gene families, leads to the smallest scallop genome. Systematic investigation of energy metabolism and biomineralization reveals a similar tendency of molecular changes underlying genome miniaturization evolution of scallops and flying birds. Our study highlights the pivotal role of genome size fluctuation in the evolution of novel lifestyles in the animal kingdom.

## Materials and methods

### Sample preparation

The Asian moon scallops (*A. pleuronectes*) were gathered from the Tung Ping Chau Bay (near Tung Chung village, Guangzhou) for whole-genome sequencing. The DNA samples were stored at the key laboratory of marine genetics and breeding, Ministry of Education, Ocean University of China (specimen code: OUC-MGB-2019-Apl-08). Dissected tissues were immediately frozen in liquid nitrogen and preserved at −80 °C for DNA extraction. The conventional hexadecyltrimethylammonium bromide (CTAB) procedure was used to extract high molecular-weight genomic DNA (gDNA) from the adductor muscle [43]. The pair-end libraries with an insert size of 350 bp were built from gDNA by utilizing Illumina genomic DNA sample preparation kits (Catalog No. 20060060, Illumina, San Diego, CA), following the manufacturer’s standard protocols, then sequenced on the Illumina Xten platform. A long-read DNA library for gDNA derived from the same individual was constructed using SMRTbell template prep kit 2.0 (Catalog No. 101-685-400, PacBio, Menlo Park, CA) on the Pacific Biosciences (PacBio) Sequel single-molecule real-time (SMRT) platform.

### Hi-C library construction and sequencing

The Hi-C library was constructed using adductor muscle tissues obtained from the same individual of *A. pleuronectes* for high-quality DNA extraction. About 5 g of the tissue sample was cut into pieces and fixed with 2% formaldehyde solution for about 20 min at room temperature. After digesting with the *Mbo*I restriction enzyme (Catalog No. R0147M, New England Biolabs, Ipswich, MA), the sticky ends of the treated fragments were repaired with biotinylated residues to form blunt-end fragments. End repair, adaptor ligation, and polymerase chain reaction (PCR) amplification were performed before sequencing. Finally, the library was sequenced with the Illumina Xten 2500 (PE150) platform.

### Genome size estimation and ***de novo*** assembly of the ***A. pleuronectes*** genome

The genome size of *A. pleuronectes* was estimated using *k*-mer frequency distribution analysis based on Illumina sequencing reads. The Trimmomatic software [44] was used to filter raw reads by trimming adaptors and discarding reads with more than 10% N bases or more than 20% low-quality bases. Then Jellyfish v2.2.5 [45] and GenomeScope v2.0 [46] were employed in measuring genome size and heterozygosity based on a 19-mer distribution. The size of the genome was estimated using the formula: genome size = *k*-mer number/*k*-mer depth.

The wtdbg2 software [47] was used to *de novo* assemble the genome using the PacBio long reads. Based on the fuzzy-Bruijn graph (FBG) algorithm, the raw reads were assembled by the wtdbg2 module, and the consensus calling of the preceding assembly was conducted with wtdbg-cns, which was also used for reducing sequencing errors. The Illumina clean reads were mapped to the assembly using Burrows-Wheeler aligner (BWA) software [48] to make sure the accuracy of the genome. Then, the Pilon software [49] was used to resolve the conflict of the assembly.

For chromosome-level scaffolding, the Hi-C sequencing technique was applied for chromosome construction for *A. pleuronectes*. The clean reads were aligned to the assembly by using BWA, and uniquely aligned reads with high mapping quality were retained for further analysis. Invalid interaction pairs were filtered by HiC-Pro software [50], then Lachesis software [51] was employed to cluster and anchor contigs to chromosomes with the agglomerative hierarchical clustering method based on the interaction matrix between sequences. The integrity and completeness of the genome assembly were evaluated by calculating the mapping rate of paired-end clean reads to the genome. For genome integrity assessment, the BUSCO v3.0.2 software [52] was applied based on the metazoan model database.

### Repeat element and ncRNA annotations

Tandem repeats were identified by Tandem Repeats Finder v4.09 [53] under default parameters, and TEs were identified via both *de novo* predictions and homology-based methods. *De novo* repetitive sequence database was constructed by RepeatModeler v1.0.11 [54], and then merged with Repbase database [55] for homology-based detection by Repeatmasker v2.1 software [56]. In addition, we conducted Kimura distance-based copy divergence analysis of four types of TEs in the intergenic regions of the six scallop genomes. For the annotation of ncRNAs in *A. pleuronectes* genome, the tRNAscan-SE software [57] was employed to annotate tRNAs, and the INFERNAL software [58] was applied to search for all other RNAs, including rRNAs, miRNAs, and snRNAs against with the Rfam database [59].

### Gene prediction and function annotation

The gene structure was predicted by integrating three methods, including homology-based search, *de novo* prediction, and RNA sequencing (RNA-seq) supporting alignment. The *de novo* prediction was conducted using Augustus [60], GlimmerHMM [61], SNAP [62], Geneid [63], and Genescan software [64]. For homology-based analysis, TBLASTN was used to align protein sequences from six bivalves (*P. yessoensis*, *C. farreri*, *A. purpuratus*, *Crassostrea gigas*, *Pinctada fucata*, and *Modiolus philippinarum*) to the *A. pleuronectes* genome assembly using TBLASTN. The accuracy of spliced alignments was then assessed by aligning homologous genome sequences to the matched proteins using GeneWise [65]. Then, the RNA-seq reads from adult tissues/organs were aligned to the assembly using Tophat software [66], and Cufflinks software [67] was employed to assemble and annotate transcripts. Finally, EVidenceModeler [68] was employed to integrate all gene models obtained from the aforementioned methods. NCBI non-redundant (Nr) protein database, Gene Ontology (GO) database [69], Kyoto encyclopedia of genes and genomes (KEGG) database [70], SwissProt [71], and InterPro were used to functionally annotate the predicted protein-coding genes.

### Transcriptome sequencing and expression profile analysis

The mantle, striated/smooth muscle, kidney, gill, gonad, and foot tissues/organs from three adult individuals were chosen for RNA-seq. Tissues/organs were dissected and immediately frozen in liquid nitrogen, followed by preservation at −80 °C until RNA extraction. Total RNA was isolated from each tissue/organ according to previously described protocols [72]. All RNA-seq libraries were prepared using the NEB next mRNA library prep kit (Catalog No. E7770, New England Biolabs) according to the manufacturer’s instructions and then sequenced on the Illumina Xten platform. The adaptors were firstly trimmed from raw reads, and reads with more than 10% N bases or more than 20% low-quality bases were filtered. STAR [73] was used to align the clean reads to the reference genome, and then FeatureCounts [74] was used to count the aligned reads to each gene. Gene expression level was represented by the count of transcripts per million (TPM), which was defined as transcripts per kilobase of exon model per million mapped reads and calculated using a custom Perl script.

### Macrosynteny and microsynteny analyses

For species with chromosome-level assemblies (*A. pleuronectes*, *P. yessoensis*, *C. farreri*, and *P. maximus*), the conservation of gene macrosynteny compared to the presumed bilaterian ancestral linkage groups (ALGs) was assessed according to the method described by Wang and colleagues [19]. For species without chromosome-level assemblies (*A. purpuratus* and *Argopecten irradians*), we employed a heuristic hierarchical method to cluster the scaffolds from the draft genome and set the tree-cutting threshold as 0.25.

For detecting conserved gene clusters among the six scallop species, we conducted *Hox* and *ParaHox* cluster identification and *de novo* searching based on genome assemblies. The *Hox* and *ParaHox* genes were identified in scallop genomes using BLASTP with an *E* value threshold of 1E−10 against known *Hox* and *ParaHox* protein sequences, and the homeobox domain was further confirmed according to the conserved domain database (CDD, http://www.ncbi.nlm.nih.gov/cdd).

### Phylogenetic tree construction and divergence time estimation

The phylogenetic tree of scallops with different lifestyles was constructed based on the available rRNA genes (12S, 16S, and 28S), including *A. pleuronectes* and other nine scallop species (*Amusium japonicum*, *A. purpuratus*, *A. opercularis*, *A. irradians*, *C. farreri*, *P. yessoensis*, *P. maximus*, *Chlamys hastata*, and *Placopecten magellanicus*), as well as one outgroup species (*Delectopecten randolphi*). The phylogenetic tree was built using BEAST2 v2.6 [75] based on the bayesian inference method.

Six scallop species were employed to construct the genome-based phylogeny, including *A. pleuronectes*, *P. yessoensis*, *C. farreri*, *A. purpuratus*, *A. irradians*, and *P. maximus*. The sequences of protein-coding genes of the six scallop species were retrieved from the MolluscDB database. Orthologous groups from the six genomes were identified using OrthoFinder v2.3.12 [76] with an *E* value of 1E−5 for BLASTP. Then, 4159 single-copy orthologs were used for multiple alignment analysis by using MAFFT software [77], and gaps were deleted using Gblocks [78]. All alignments were combined into a supermatrix to construct a phylogenetic tree using RAxML software [79] with 1000 rapid bootstrap analyses. Finally, the divergence time of species was estimated based on the MCMCTree module in the PAML package [80] combined with a molecular clock model. Two reference divergence time points (500−550 MYA between *Lottia* and *Crassostrea*; > 330 MYA between *Patinopecten* and *Crassostrea*) retrieved from the TimeTree database [81] were used to calibrate divergence dates of other nodes.

### TE-related analysis

To investigate the dynamics of TE activities during the evolution of scallops, we employed the free-ratio model of PAML software to calculate the nucleotide substitution rates [80], and RepeatMasker was used to calculate TE sequence divergences from consensus sequences. The substitution level *K* of TEs was calculated according to the Jukes-Cantor formula *K* = 3/4Ln(1 − 4d/3), in which *K* is the genetic distance and d represents the number of observed substitutions normalized by the sequence length. We employed the formula *T* = *K*/2*r* [82] to estimate insertion time of TEs, in which *r* represents the nucleotide substitution rate for each scallop.

To categorize the gene families, six scallop species were scanned using the HMM approach on known Pfam functional domains. A protein containing multiple copies of a domain was only counted once. The gene family expansion/contraction analysis for *A. pleuronectes* was conducted by comparing the average domain counts in the other five scallop species using the Fisher test in R. And the gene families with *P* < 0.05 were identified as significantly contracted or expanded ones.

### Analysis of size-reducing genes in the scallop *A. pleuronectes* and the bird *C. anna*

Single-copy orthologous genes among six scallop species were identified using OrthoFinder v2.3.12 [76] with a threshold *E* value of 1E−5 for BLASTP. To identify size-reducing genes in the scallop *A. pleuronectes*, the length of paired single-copy orthologous genes in six scallop species were standardized using gene length divided by the genic region of the genome. Genes with the relative length value in *A. pleuronectes* smaller than the average value of the other five less-active scallop species were defined as size-reducing genes. Similarly, single-copy orthologous genes from the four tetrapods were identified using OrthoFinder with an *E* value of 1E−5. To identify size-reducing genes in the bird *C. anna*, the length of paired single-copy orthologous genes in four tetrapods were standardized using gene length divided by the genic region of the genome. Genes with a relative length value in *C. anna* smaller than the average value of the other three non-flying tetrapods were defined as size-reducing genes. Genome assemblies of four tetrapods were downloaded from the NCBI genome database, including *C. anna* (NCBI genome: bCalAnn1_v1.p), *C. serpentina* (NCBI genome: ASM1885937v1), *X. laevis* (NCBI genome: Xenopus_laevis_v10.1), and *M. musculus* (NCBI genome: GRCm39).

Size-reducing genes in *A. pleuronectes* and *C. anna* were employed for KEGG enrichment analysis by EnrichPipeline [83], respectively. Remarkable enrichment of KEGG pathways (*P* < 0.05) was shown by bubble plot. We compared the expression levels (TPM) of size-reducing genes enriched in the oxidative phosphorylation pathway in striated muscles between *A. pleuronectes* and the other two scallop species (*P. yessoensis* and *C. farreri*). The complex colors of the oxidative phosphorylation pathway plots represent the up-regulation degree for genes in *A. pleuronectes* according to the ratio of the TPM of a gene in *A. pleuronectes* divided by the average TPM of the gene in the other two less-active scallop species. In addition, we downloaded the adductor muscle transcriptome data of *A. purpuratus* from NCBI (SRA: SRR6849474), and compared it with mixed transcriptome data of striated and smooth muscles of *A. pleuronectes*, *P. yessoensis*, and *C. farreri*. Transcriptome analyses of basal transcription factors and aminoacyl-tRNA biosynthesis pathways were performed among four scallop species (*A. pleuronectes*, *P. yessoensis*, *C. farreri*, and *A. purpuratus*). Transcriptome data of *C. farreri* and *P. yessoensis* were retrieved from studies of Wang et al. [19] and Li et al. [20], and nine transcriptome data of *A. purpuratus* were obtained from NCBI (SRA: SRR6849473, SRR6849474, SRR6849475, SRR7993930, SRR7993931, SRR7993936, SRR7993937, SRR7993946, and SRR7993947).

### Biomineralization-related gene analysis

The identification of putative biomineralization-related genes was conducted by aligning to well-known Pfam protein domains according to the bivalve biomineralization toolbox [40]. Normalized domain number in the three scallop species (*A. pleuronectes*, *P. yessoensis*, and *C. farreri*) was compared by ggboxplot package in R, and a comparison of expression levels of biomineralization-related genes in mantle was shown in the bar plot. To construct the co-expression gene network, the R package of WGCNA [84] was employed based on the adult transcriptome data of three scallop species including *A. pleuronectes*, *P. yessoensis*, and *C. farreri*. The modules that enriched genes specifically highly expressed in the mantle tissue was considered a biomineralization-related module. The connection strength for genes in each module was represented by the intramodular connectivity value (*K*_within_), which is an important factor for determining hub genes. Then, we used Cytoscape software to visualize the top 40 hub genes in the biomineralization-related modules of the three scallop species. OrthoFinder was used for detecting single-copy orthologous genes in the three scallop species, and relative gene lengths were employed to determine length-increasing genes in *A. pleuronectes* in the biomineralization-related module by comparing to the other two scallop species. And the results were shown by a heatmap plot in R.

For the analysis of bone formation-related genes, we collected marker genes of immature cartilage, mature cartilage, and bone according to the previous study [42]. Then, we verified the expression pattern of bone marker genes by the expression profile of adult organs/tissues in *G. gallus*. RNA-seq data of *G. gallus* were downloaded from NCBI (SRA: SRR594500–SRR594526, SRR16693970–SRR16693975, and SRR16693980–SRR16693982).

## Code availability

The code developed for the study has been submitted to BioCode at the National Genomics Data Center (NGDC), Beijing Institute of Genomics (BIG), Chinese Academy of Sciences (CAS) / China National Center for Bioinformation (CNCB) (BioCode: BT007309), which is publicly accessible at https://ngdc.cncb.ac.cn/biocode/tools/BT007309.

## Data availability

The *A. pleuronectes* scallop genome project has been deposited in NCBI (BioProject: PRJNA797172). The genome sequencing data have been uploaded to Sequence Read Archive (SRA: SRR17752961–SRR17752967, SRR17752978, SRR17752989, and SRR17752990). The Illumina sequencing data of 20 transcriptomes for adult tissues/organs have also been uploaded to SRA (SRA: SRR17752968–SRR17752977 and SRR17752979–SRR17752988). The assembled data of *A. pleuronectes* have also been submitted to Genome Warehouse [85] at the NGDC, BIG, CAS / CNCB (GWH: GWHBJCZ00000000), and are publicly accessible at https://ngdc.cncb.ac.cn/gwh. The raw sequencing data have also been deposited in the Genome Sequence Archive [86] at the NGDC, BIG, CAS / CNCB (GSA: CRA006995), and are publicly accessible at https://ngdc.cncb.ac.cn/gsa.

## CRediT author statement

**Yuli Li:** Data curation, Methodology, Formal analysis, Writing - review & editing. **Yaran Liu:** Software, Formal analysis, Data curation, Methodology, Writing - original draft. **Hongwei Yu:** Formal analysis, Methodology. **Fuyun Liu:** Software, Data curation. **Wentao Han:** Formal analysis. **Qifan Zeng:** Data curation. **Yuehuan Zhang:** Data curation. **Lingling Zhang:** Writing - review & editing. **Jingjie Hu:** Writing - review & editing. **Zhenmin Bao:** Conceptualization, Project administration, Writing - review & editing. **Shi Wang:** Conceptualization, Project administration, Writing - review & editing. All authors have read and approved the final manuscript.

## Competing interests

The authors declare that they have no competing interests.

## Supplementary material

Supplementary Table S1 **Summarized information for 9 scallop species**

Supplementary Table S2 **Distance in one swimming burst and genome size of 5 scallops**

Supplementary Table S3 **Gene sequences for phylogenetic tree of 10 scallop species**

Supplementary Table S4 **Summary of genome sequencing data of *A. pleuronectes***

Supplementary Table S5 **Summary of transcriptome sequencing data of *A. pleuronectes***

Supplementary Table S6 **Summary of the chromosome-level genome assembly and annotations for *A. pleuronectes***

Supplementary Table S7 **Summary of assembled 19 chromosomes of *A. pleuronectes***

Supplementary Table S8 **BUSCO evaluation of genome quality of *A. pleuronectes***

Supplementary Table S9 **Summary of gene functional annotation for *A. pleuronectes* genome**

Supplementary Table S10 **Annotations of non-coding RNA in the *A. pleuronectes* genome**

Supplementary Table S11 **Summary of repeat sequences in the *A. pleuronectes* genome**

Supplementary Table S12 **Microsynteny gene cluster in six scallop genomes**

Supplementary Table S13 **Summary of the six scallop genome assemblies**

Supplementary Table S14 **Pfam list of the bivalve biomineralization toolbox**
