# Supplementary material for: Adaptive Bird-like Genome Miniaturization During the Evolution of Scallop Swimming Lifestyle
Source: Genomics Proteomics Bioinformatics. 2022 Jul 26;20(6):1066–77. doi: 10.1016/j.gpb.2022.07.001 (PMC10225492; doi:10.1016/j.gpb.2022.07.001)
Supplement: Supplementary Table S1 — Summarized information for 9 scallop species [file mmc1.docx]

**Table S1 Summarized information for 9 scallop species**

| **Species** | **Common name** | **Genome size (Gb)** | **Lifestyle** | **Latitude^a^** |
| --- | --- | --- | --- | --- |
| *A. japonicum* | Japanese Moon scallop | 0.73 | Gliding | 31°N − 21°S |
| *A. pleuronectes* | Asian Moon scallop | 0.67 | Gliding | 31°N − 21°S |
| *A. purpuratus* | Peruvian scallop | 1.1 | Free living | 5°S − 42°S |
| *A. irradians* | Bay scallop | 1.2 | Free living | 45°N − 8°N |
| *P. yessoensis* | Yesso scallop | 1.4 | Recessing | 56°N − 35°N |
| *P. maximus* | King scallop | 1.42 | Recessing | 23°N − 66°N |
| *A. opercularis* | Queen scallop | 1.12 | Byssal attach | 68°N − 15°N |
| *C. farreri* | Chinese scallop | 1.2 | Byssal attach | 23.5°N − 40°N |
| *C. hastata* | Spiny scallop | 1.64 | Byssal attach | 23°N − 66°N |

*Note*: ^a^, The latitude data were derived from the Sealifebase database (https://www.sealifebase.ca/).
