# Supplementary material for: Adaptive Bird-like Genome Miniaturization During the Evolution of Scallop Swimming Lifestyle
Source: Genomics Proteomics Bioinformatics. 2022 Jul 26;20(6):1066–77. doi: 10.1016/j.gpb.2022.07.001 (PMC10225492; doi:10.1016/j.gpb.2022.07.001)
Supplement: Supplementary Table S2 — Distance in one swimming burst and genome size of 5 scallops [file mmc2.docx]

**Table S2 Distance in one swimming burst and genome size of 5 scallop**

| **Species** | **Genome size (Gb)** | **Distance (m)** |
| --- | --- | --- |
| *A. pleuronectes* | 0.67 | >10 |
| *A. irradians* | 1.2 | 7.6 |
| *A. opercularis* | 1.1 | 6.6 |
| *P. maximus* | 1.42 | 3 |
| *P. magellanicus* | 2.1 | 4.3 |
