# Supplementary material for: Adaptive Bird-like Genome Miniaturization During the Evolution of Scallop Swimming Lifestyle
Source: Genomics Proteomics Bioinformatics. 2022 Jul 26;20(6):1066–77. doi: 10.1016/j.gpb.2022.07.001 (PMC10225492; doi:10.1016/j.gpb.2022.07.001)
Supplement: Supplementary Table S3 — Gene sequences for phylogenetic tree of 10 scallop species [file mmc3.docx]

**Table S3 Gene sequences for phylogenetic tree of 10 scallop species**

| **Species** | **12S** | **16S** | **28S** |
| --- | --- | --- | --- |
| *A. japonicum* | HM622706 | KJ000149 | HM622709 |
| *A. pleuronectes* | EU379415 | EU379469 | HM630508 |
| *A. purpuratus* | EU379417 | EU379471 | HM630495 |
| *C. opercularis* | EU379408 | EU379462 | HM630527 |
| *A. irradians* | EU379392 | EU379446 | HM622700 |
| *C. farreri* | HM622681 | HM622678 | HM622680 |
| *P. yessoensis* | FJ263640 | HM630384 | FJ263660 |
| *P. maximus* | EU379400 | EU379454 | HM630545 |
| *C. hastata* | FJ263639 | FJ263648 | FJ263658 |
| *P. magellanicus* | FJ263638 | FJ263647 | FJ263657 |
| *D. randolphi* | HM630488 | HM630489 | HM630491 |
