# Supplementary material for: Adaptive Bird-like Genome Miniaturization During the Evolution of Scallop Swimming Lifestyle
Source: Genomics Proteomics Bioinformatics. 2022 Jul 26;20(6):1066–77. doi: 10.1016/j.gpb.2022.07.001 (PMC10225492; doi:10.1016/j.gpb.2022.07.001)
Supplement: Supplementary Table S4 — Summary of genome sequencing data of A. pleuronectes [file mmc4.docx]

**Table S4 Summary of genome sequencing data of *A. pleuronectes***

| **Library type** | **Insert size** | **Total data (Gb)** | **Mean reads length (bp)** | **Sequence depth (*×*)** |
| --- | --- | --- | --- | --- |
| Illumina reads | 350 bp | 99.87 | 150 | 149.71 |
| Pacbio reads | 20 kb | 83.9 | - | 147.78 |
| Hi-C reads | - | 91.53 | - | 161.22 |
| Total | - | 275.3 | - | 458.71 |

*Note*: Genome size used to calculate sequencing coverage were 667.07 M, which is estimated by the Kmer-based method.
