# Supplementary material for: Adaptive Bird-like Genome Miniaturization During the Evolution of Scallop Swimming Lifestyle
Source: Genomics Proteomics Bioinformatics. 2022 Jul 26;20(6):1066–77. doi: 10.1016/j.gpb.2022.07.001 (PMC10225492; doi:10.1016/j.gpb.2022.07.001)
Supplement: Supplementary Table S5 — Summary of transcriptome sequencing data of A. pleuronectes [file mmc5.docx]

**Table S5 Summary of transcriptome sequencing data of *A. pleuronectes***

| **Tissue/Organs** | **Number of paired-end reads** | **Unique mapping rate (%)** | **Accession number** |
| --- | --- | --- | --- |
| Striated_muscle_1 | 20,702,801 | 93.13 | SRR17752985 |
| Striated_muscle_2 | 24,474,981 | 92.78 | SRR17752984 |
| Striated_muscle_3 | 18,751,308 | 93.58 | SRR17752983 |
| Smooth_muscle_1 | 20,466,852 | 92.85 | SRR17752988 |
| Smooth_muscle_2 | 17,825,553 | 93.17 | SRR17752987 |
| Smooth_muscle_3 | 19,421,244 | 92.94 | SRR17752986 |
| Gill_1 | 22,964,119 | 91.23 | SRR17752982 |
| Gill_2 | 20,775,185 | 91.84 | SRR17752981 |
| Gill_3 | 20,581,010 | 90.75 | SRR17752980 |
| Kidney_1 | 19,043,680 | 90.72 | SRR17752979 |
| Kidney_2 | 20,900,308 | 90.85 | SRR17752977 |
| Kidney_3 | 17,282,937 | 89.07 | SRR17752976 |
| Mantle_1 | 19,512,560 | 91.33 | SRR17752972 |
| Mantle_3 | 17,519,824 | 91.27 | SRR17752971 |
| Gonad_1 | 18,714,985 | 91.38 | SRR17752975 |
| Gonad_2 | 21,733,990 | 89.84 | SRR17752974 |
| Gonad_3 | 19,418,721 | 89.75 | SRR17752973 |
| Foot_1 | 17,153,306 | 90.97 | SRR17752970 |
| Foot_2 | 18,802,154 | 91.84 | SRR17752969 |
| Foot_3 | 19,212,556 | 90.89 | SRR17752968 |
