# Supplementary material for: Adaptive Bird-like Genome Miniaturization During the Evolution of Scallop Swimming Lifestyle
Source: Genomics Proteomics Bioinformatics. 2022 Jul 26;20(6):1066–77. doi: 10.1016/j.gpb.2022.07.001 (PMC10225492; doi:10.1016/j.gpb.2022.07.001)
Supplement: Supplementary Table S6 — Summary of the chromosome-level genome assembly and annotations for A. pleuronectes [file mmc6.docx]

**Table S6 Summary of the chromosome-level genome assembly and annotations for *A. pleuronectes***

| **Genome assembly** | **Value** |
| --- | --- |
| Total size (bp) | 626,631,671 |
| Contig N50 size (bp) | 2,641,118 |
| Scaffold N50 size (bp) | 34,611,732 |
| Average scaffold length | 2,175,804 |
| Number of chromosomes | 19 |
| Length of chromosomes (bp) | 609,550,000 |
| Integration efficiency of Hi-C mapping (%) | 97.27% |
| GC content (%) | 35.52% |
