# Supplementary material for: Adaptive Bird-like Genome Miniaturization During the Evolution of Scallop Swimming Lifestyle
Source: Genomics Proteomics Bioinformatics. 2022 Jul 26;20(6):1066–77. doi: 10.1016/j.gpb.2022.07.001 (PMC10225492; doi:10.1016/j.gpb.2022.07.001)
Supplement: Supplementary Table S7 — Summary of assembled 19 chromosomes of A. pleuronectes [file mmc7.docx]

**Table S7 Summary of assembled 19 chromosomes of *A. pleuronectes***

| **Chromosomes** | **Length (bp)** | **Number of contigs** |
| --- | --- | --- |
| chr1 | 42,155,244 | 24 |
| chr2 | 41,476,497 | 19 |
| chr3 | 39,976,771 | 17 |
| chr4 | 38,742,912 | 18 |
| chr5 | 36,971,373 | 18 |
| chr6 | 35,250,860 | 18 |
| chr7 | 35,049,405 | 22 |
| chr8 | 34,611,732 | 15 |
| chr9 | 31,969,165 | 18 |
| chr10 | 34,783,639 | 18 |
| chr11 | 32,595,778 | 15 |
| chr12 | 31,443,528 | 22 |
| chr13 | 29,501,951 | 18 |
| chr14 | 29,551,105 | 18 |
| chr15 | 26,626,294 | 18 |
| chr16 | 23,898,553 | 16 |
| chr17 | 24,042,565 | 25 |
| chr18 | 20,773,935 | 26 |
| chr19 | 20,128,693 | 30 |
