# Supplementary material for: Adaptive Bird-like Genome Miniaturization During the Evolution of Scallop Swimming Lifestyle
Source: Genomics Proteomics Bioinformatics. 2022 Jul 26;20(6):1066–77. doi: 10.1016/j.gpb.2022.07.001 (PMC10225492; doi:10.1016/j.gpb.2022.07.001)
Supplement: Supplementary Table S8 — BUSCO evaluation of genome quality of A. pleuronectes [file mmc8.docx]

**Table S8 BUSCO evaluation of genome quality of *A. pleuronectes***

| **BUSCO** | **Percentage (%)** |
| --- | --- |
| Complete BUSCOs | 95.0 |
| Complete and single-copy BUSCOs | 93.9 |
| Complete duplicated BUSCOs | 1.1 |
| Fragmented BUSCOs | 1.5 |
| Missing BUSCOs | 3.5 |

*Note*: BUSCO, .
