# Supplementary material for: Adaptive Bird-like Genome Miniaturization During the Evolution of Scallop Swimming Lifestyle
Source: Genomics Proteomics Bioinformatics. 2022 Jul 26;20(6):1066–77. doi: 10.1016/j.gpb.2022.07.001 (PMC10225492; doi:10.1016/j.gpb.2022.07.001)
Supplement: Supplementary Table S9 — Summary of gene functional annotation for A. pleuronectes genome [file mmc9.docx]

**Table S9 Summary of gene functional annotation for *A. pleuronectes* genome**

|  | **Number** | **Percentage (%)** |
| --- | --- | --- |
| Total | 24,359 | - |
| Swissprot | 16,432 | 67.46 |
| Nr | 23,126 | 94.94 |
| KEGG | 18,356 | 75.36 |
| InterPro | 18,412 | 75.59 |
| GO | 11,853 | 48.66 |
| Pfam | 14,956 | 61.40 |
| Annotated | 23,225 | 95.34 |

*Note*: KEGG, ; GO, ; Nr, ; InterPro, ; Pfam .
