# Supplementary material for: Adaptive Bird-like Genome Miniaturization During the Evolution of Scallop Swimming Lifestyle
Source: Genomics Proteomics Bioinformatics. 2022 Jul 26;20(6):1066–77. doi: 10.1016/j.gpb.2022.07.001 (PMC10225492; doi:10.1016/j.gpb.2022.07.001)
Supplement: Supplementary Table S10 — Annotations of non-coding RNA in the A. pleuronectes genome [file mmc10.docx]

**Table S10 Annotations of non-coding RNA in the *A. pleuronectes* genome**

| **Type** | | **Copy number** | **Average length (bp)** | **Total length (bp)** | **Percentage of genome (%)** |
| --- | --- | --- | --- | --- | --- |
| rRNA | rRNA | 146 | 167.79 | 24,497 | 0.37 |
|  | 18S | 26 | 313.46 | 8150 | 0.12 |
|  | 28S | 56 | 200.12 | 11,207 | 0.17 |
|  | 5.8S | 3 | 153.67 | 461 | 0.01 |
|  | 5S | 61 | 76.7 | 4679 | 0.07 |
| miRNA | | 302 | 112.9 | 34,096 | 0.51 |
| snRNA | snRNA | 185 | 147.66 | 27,318 | 0.41 |
|  | CD-box | 34 | 101.97 | 3467 | 0.05 |
|  | HACA-box | 24 | 194.92 | 4678 | 0.07 |
|  | splicing | 112 | 144.68 | 16,204 | 0.24 |
|  | scaRNA | 9 | 162.78 | 1465 | 0.02 |
|  | Unknown | 6 | 250.67 | 1504 | 0.02 |
| tRNA | | 908 | 74.48 | 67,625 | 1.02 |

*Note*: rRNA, ; snRNA, ;CD-box, ; HACA-box, ; scaRNA, .
