# Supplementary material for: Adaptive Bird-like Genome Miniaturization During the Evolution of Scallop Swimming Lifestyle
Source: Genomics Proteomics Bioinformatics. 2022 Jul 26;20(6):1066–77. doi: 10.1016/j.gpb.2022.07.001 (PMC10225492; doi:10.1016/j.gpb.2022.07.001)
Supplement: Supplementary Table S11 — Summary of repeat sequences in the A. pleuronectes genome [file mmc11.docx]

**Table S11 Summary of repeat sequences in the *A. pleuronectes* genome**

|  | **Length (bp)** | **Percentage of genome (%)** |
| --- | --- | --- |
| DNA | 22,456,109 | 3.58 |
| LINE | 18,404,365 | 2.94 |
| SINE | 9,693,816 | 1.55 |
| LTR | 9,301,548 | 1.48 |
| Tandem repeat | 136,005,223 | 21.70 |
| Unknown | 70,029,433 | 11.18 |
| Total | 243,782,685 | 38.90 |

*Note*: LINE, ; SINE, ; LTR, .
