# Supplementary material for: Adaptive Bird-like Genome Miniaturization During the Evolution of Scallop Swimming Lifestyle
Source: Genomics Proteomics Bioinformatics. 2022 Jul 26;20(6):1066–77. doi: 10.1016/j.gpb.2022.07.001 (PMC10225492; doi:10.1016/j.gpb.2022.07.001)
Supplement: Supplementary Table S12 — Microsynteny gene cluster in six scallop genomes [file mmc12.docx]

**Table S12 Microsynteny gene cluster in six scallop genomes**

| **Species** | ***Taxilin*** | ***HECA*** | ***SYAP1*** | ***PSMG1*** | ***ZMPSTE24*** |
| --- | --- | --- | --- | --- | --- |
| *A. pleuronectes* | evm.model.ctg43_pilon.26.4 | evm.model.ctg43_pilon.25 | evm.model.ctg43_pilon.22 | evm.model.ctg43_pilon.21 | evm.model.ctg25_pilon.102 |
| *A. irradians* | evm.model.Contig429.57 | evm.model.Contig429.58 | evm.model.Contig429.60 | evm.model.Contig429.61 | evm.model.Contig352.63 |
| *A. purpuratus* | evm.model.scaffold_848.49 | evm.model.scaffold_848.48 | evm.model.scaffold_848.47 | evm.model.scaffold_848.46 | evm.model.scaffold_509.6 |
| *P. yessoensis* | evm.model.scaffold7763.29.5 | evm.model.scaffold7763.28 | evm.model.scaffold7763.24 | evm.model.scaffold7763.20 | evm.model.scaffold2995.22 |
| *C. farreri* | evm.model.scaffold14127.30 | evm.model.scaffold14127.31 | evm.model.scaffold14127.33 | evm.model.scaffold14127.34 | evm.model.scaffold51905.37 |
| *P. maximus* | evm.model.000064F_PILON.61.4;evm.model.000101F_PILON.79.4 | evm.model.00064F_PILON.62;evm.model.000101F_PILON.76 | evm.model.000064F_PILON.63;evm.model.000101F_PILON.74 | evm.model.000064F_PILON.64;evm.model.000101F_PILON.73 | evm.model.000074F_PILON.37 |
