# Supplementary material for: Adaptive Bird-like Genome Miniaturization During the Evolution of Scallop Swimming Lifestyle
Source: Genomics Proteomics Bioinformatics. 2022 Jul 26;20(6):1066–77. doi: 10.1016/j.gpb.2022.07.001 (PMC10225492; doi:10.1016/j.gpb.2022.07.001)
Supplement: Supplementary Table S13 — Summary of the six scallop genome assemblies [file mmc13.docx]

**Table S13 Summary of the six scallop genome assemblies**

| **Species** | ***A. pleuronectes*** | ***A. irradians*** | ***A. purpuratus*** | ***P. yessoensis*** | ***C. farreri*** | ***P. maximus*** |
| --- | --- | --- | --- | --- | --- | --- |
| Genome size (Mb) | 626.63 | 835.7 | 724.78 | 1000.60 | 816.5 | 1060.24 |
| Gene region (%) | 56.95 | 42.75 | 44.18 | 42.32 | 43.20 | 36.58 |
| Intergenic region (%) | 43.05 | 57.25 | 55.82 | 57.68 | 56.80 | 63.42 |
| Scaffold N50 (Mb) | 34.61 | 1.53 | 1.02 | 0.83 | 0.56 | 50.63 |
| Total size of genes (Mb) | 356.84 | 357.25 | 320.2 | 423.42 | 352.73 | 387.86 |
| Total size of exons (Mb) | 63.7 | 54.96 | 57.22 | 52.18 | 51.76 | 56.41 |
| Total size of introns (Mb) | 293.47 | 302.63 | 263.31 | 371.57 | 301.29 | 331.8 |
| Number of coding-genes | 24,359 | 26,777 | 26,256 | 24,738 | 28,602 | 26,995 |
| GC content (%) | 35.52 | 35.01 | 34.52 | 33.12 | 33.99 | 37.67 |
| TE size (Mb) | 129.9 | 280.63 | 219.9 | 253.75 | 207.55 | 342.35 |
| TE content of genome (%) | 20.73 | 33.58 | 30.34 | 25.36 | 25.42 | 32.29 |
| TE content of intergenic region (%) | 6.58 | 10.33 | 9.58 | 9.23 | 9.62 | 9.74 |
| TE content of exonic region (%) | 7.08 | 6.39 | 6.71 | 4.54 | 4.24 | 6.41 |
| TE content of intronic region (%) | 24.82 | 34.97 | 30.79 | 28.93 | 28.43 | 29.63 |
| Tandem repeat size (Mb) | 136.24 | 126.41 | 83.24 | 184.82 | 92.59 | 380.1 |
| Tandem repeat content of genome (%) | 21.74 | 15.13 | 11.49 | 18.47 | 11.34 | 35.85 |
| Tandem repeat content of intergenic region (%) | 5.46 | 9.58 | 8.21 | 7.78 | 5.78 | 31.71 |
| Tandem repeat content of exonic region (%) | 12.75 | 9.7 | 7.19 | 11.54 | 11.57 | 18.54 |
| Tandem repeat content of intronic region (%) | 38.69 | 24.9 | 17.46 | 35.36 | 19.87 | 47.18 |

*Note*: TE .
