# Supplementary material for: Adaptive Bird-like Genome Miniaturization During the Evolution of Scallop Swimming Lifestyle
Source: Genomics Proteomics Bioinformatics. 2022 Jul 26;20(6):1066–77. doi: 10.1016/j.gpb.2022.07.001 (PMC10225492; doi:10.1016/j.gpb.2022.07.001)
Supplement: Supplementary Table S14 — Pfam list of the bivalve biomineralization toolbox [file mmc14.docx]

**Table S14 Pfam list of the bivalve biomineralization toolbox related genes**

| **Pfam domain** | **Description** | **Pfam ID** |
| --- | --- | --- |
| EGF | Calcium-binding EGF domain | PF07645 |
| Cu_monooxygenase | Copper type II ascorbate-dependent monooxygenase | PF03712; PF01082 |
| WAP | WAP-type (Whey Acidic Protein) | PF00095 |
| Kunitz | Kunitz/Bovine pancreatic trypsin inhibitor domain | PF00014 |
| FKBP | KBP-type peptidyl-prolyl cis-trans isomerase | PF00254 |
| Kazal | Kazal-type serine protease inhibitor domain | PF00050; PF07648 |
| P-loop | P-loop domain | PF07693 |
| NTPase | NTPase | PF03266 |
| Glycosyl_hydrolase | Glycosyl hydrolases family | PF00704; PF00728 |
| PPlase | peptidyl-prolyl cis-trans isomerase | PF00160 |
| CA | Carbonic anhydrase | PF00194 |
| Chitin | Chitin binding Peritrophin-A domain | PF01607 |
| macroglobulin | alpha-2-macroglobulin | PF00207 |
| Tyrosinase | tyrosinase | PF00264 |
| IG | immunoglobulin | PF13927 |
| VWA | Von Willebrand factor type A | PF00092 |
| FN3 | fibronectin-3 | PF00041 |
| C1q | complement component | PF00386 |
| TIMP | tissue inhibitor of metalloproteinase | PF00965 |
